# Uncovering the Chemosensory System of a Subterranean Termite, *Odontotermes formosanus* (Shiraki) (Isoptera: Termitidae): Revealing the Chemosensory Genes and Gene Expression Patterns

**DOI:** 10.3390/insects14110883

**Published:** 2023-11-15

**Authors:** Rana Muhammad Kaleem Ullah, Bao Jia, Sheng Liang, Aatika Sikandar, Fukun Gao, Haiyan Wu

**Affiliations:** 1 State Key Laboratory for Conservation and Utilization of Subtropical Agro-Bioresources, Guangxi Key Laboratory of Agric-Environment and Agric-Products Safety, College of Agriculture, Guangxi University, Nanning 530004, China; ranakaleem193@gmail.com (R.M.K.U.); aatikasikandar@gxu.edu.cn (A.S.); 2117392012@st.gxu.edu.cn (F.G.); 2 Nanning Institute of Termite Control, Nanning 530023, China; 331615265@163.com (B.J.); 13977126270@163.com (S.L.)

**Keywords:** olfactory system, chemosensory genes, *Odontotermes formosanus*, social behavior

## Abstract

**Simple Summary:**

In order to sense the numerous chemical signals from their surroundings, insects have developed complex olfactory systems and olfactory mechanisms involving a wide range of chemosensory genes. Olfaction therefore plays an integral role in directing and regulating all insect behaviors and activities. Termites possess significant ecological significance due to their eusocial nature; that is, they use intricate chemical communication mechanisms to coordinate colony organization and regulate social behavior. In this study, a transcriptomic analysis of *Odontotermes formosanus* workers was performed to uncover candidate chemosensory genes. Forty-two candidate chemosensory genes were identified, and the relative expression profiles of these candidate genes were investigated. This study reveals new directions for the study of chemosensory genes and uncovers the molecular underpinnings of the functional olfactory system in termites, which will lead to the green pest management of termites in the future.

**Abstract:**

Termites are eusocial insects. Chemical signals between colony members are crucial to the smooth running of colony operations, but little is known about their olfactory system and the roles played by various chemosensory genes in this process. Chemosensory genes are involved in basic olfactory perception in insects. *Odontotermes formosanus* (Shiraki) is one of the most damaging pests to agricultural crops, forests, and human-made structures. To better understand the olfactory system and the genes involved in olfactory processing in *O. formosanus*, we produced a transcriptome of worker termites. In this study, we identified 13 *OforOBPs*, 1 *OforCSP*, 15 *OforORs*, 9 *OforGRs*, and 4 *OforSNMPs*. Multiple sequence alignments were used in the phylogenetic study, which included data from other termite species and a wide variety of insect species. Moreover, we also investigated the mRNA expression levels using qRT-PCR. The significantly high expression levels of *OforCSP1*, *OforOBP2*, *OforOR1*, and *OforSNMP1* suggest that these genes may play important roles in olfactory processing in termite social behavior, including caste differentiation, nestmate and non-nestmate discrimination, and the performance of colony operations among members. Our research establishes a foundation for future molecular-level functional studies of chemosensory genes in *O. formosanus*, which might lead to the identification of novel targets for termite integrated pest management.

## 1. Introduction

Termites are notoriously destructive pests that feed on non-cellulose materials and have been linked to damage to buildings, forests, agricultural crops, traffic infrastructure, cable bridges, and dams [1,2,3]. Termites have a broad distribution range, and the severity of their damage is more or less due to their complicated eating patterns. Severe consequences might include the collapse of structures and endangerment of human life, resulting in irreparable damage. The Yangtze River region has suffered catastrophic termite devastation, and in 1992, there were 476 species of termites known to exist in China. China had already seen yearly termite damage of more than CNY 2 billion at that point. Moreover, in the United States, termite damage caused an estimated loss of USD 5 billion in 1998 [4]. *Odontotermes formosanus* (Shiraki) is a species of subterranean termite that belongs to the family Termitidae in the insect order Isoptera. *O. formosanus* is widely distributed across Southeast Asia, as well as in China, Vietnam, Japan, India, Burma, and Thailand [5]. Besides invading plantations and agricultural crops, *O. formosanus* may damage pipes within earthen dikes by constructing enormous underground cavities, which can cause the dikes and dams to collapse [6]. Workers mainly eat bark and roots, preferably in humid circumstances [6]. *O. formosanus* nests are so well concealed that it may be challenging to recognize the damage that this species causes; nonetheless, by the time the nest is identified, a large amount of damage will have already been done. This pest can damage more than 100 plant species, notably maple, cedar, magnolia, and eucalyptus [7]. In the region of Southeast Asia from the Malay Peninsula, researchers discovered fascinating occurrences of ring barking in addition to root debarking in dipterocarp saplings. *Odontotermes* was the most prevalent among the various genera of termites discovered on or around the main roots of the saplings [8]. 

In insects, the antennae are usually the primary and most important olfactory organs for perceiving and interpreting the chemical stimuli that are present in their surroundings. Chemical signals that are sent out within the same group of species, to natural enemies, and even to host plants can be picked up using the antennal sensilla of the insect and lead to a variety of decisions regarding behavior, such as the selection of a mate, the determination of the precise spot of a host, and determining the presence of natural predators [9]. Among social insects, another layer is added to chemosensory genomics since their interactions are complicated and chemical-based. In addition to organizing the colony’s operations, social insects must communicate with a huge number of individuals [10]. Social insects recognize nestmates by aligning chemical signals on the individual’s surfaces using another individual’s nestmate discriminator neural template, which is experience-derived. Researchers provide evidence that, in ants, *Cataglyphis niger* recognizes its nestmates using hydrocarbons [11]. 

Insects use three different types of receptors, including odorant receptors (ORs), ionotropic receptors (IRs), and gustatory receptors (GRs) during the chemoreceptive process [12,13]. In the context of chemoreceptor research, ORs have received the greatest attention. OR proteins, which have seven transmembrane domains, usually appear on the dendrite region of odorant sensory neurons (OSNs) [14,15]. The absence of Orco impairs the effectiveness of each of the ORs [16,17]. Furthermore, sensory neuron membrane proteins (SNMPs) have been demonstrated to be found in moths on their OSN dendritic membranes [18]. SNMPs are members of the CD36 protein family that have been discovered in a variety of insect species [18,19,20]. Odorant binding proteins (OBPs) and chemosensory proteins (CSPs) are two important insect transporters. Both proteins are hydrophilic, having spherical forms and pockets harboring hydrophobic compounds [21]. An alpha helical structure and disulfide bonds (two in CSPs [22] and three in the case of OBPs [23,24]), are found in these proteins, while in the case of OBPs, a larger or smaller number (C-plus and C-minus OBPs) of cysteines can be present [25,26,27,28]. It is believed that the vast majority of OBPs and CSPs have a role in the perception of pheromones, including chemical compounds [29]. Pest control technology based on the olfactory manipulation of behavior is a green pest control method that offers direct control over the behavior of target pests, and has many possible uses [30].

Genomic and transcriptomic techniques have been utilized to identify chemosensory genes in many types of insects [14,15,31]. Recently, there has been an increase in interest in investigations of genomics along with gene expression studies in termites [32,33,34,35,36]. The latest developments in the research on the functional genomics of termites have been useful in precisely understanding the distinctive and fascinating aspects of termite biology [37], like understanding aggression and differences between castes on a genetic level [38]. These developments have allowed us to comprehend their behavioral and evolutionary adaptability in many biological circumstances involving eusociality, and also to understand chemosensory systems [28,39,40,41,42,43,44,45]. Nonetheless, despite their great ecological, evolutionary, as well as economic importance, subterranean termites are a genetic and genomically unexplored group of insects [46].

The research on termites is mainly focused on chemical ecology, as well as pheromone biology; however, there are only six species of termites in which the chemosensory genes have been identified, three of which are *Zootermopsis nevadensis* [42], *Cryptotermes secundus* [47], and *Reticulitermes speratus* [48], having 85, 42, and 22 ORs in their genomes, respectively. In another study, the chemosensory genes of three termite species from three distinct lineages, *Neotermes cubanus*, *Prorhinotermes simplex*, and *Inquilinitermes inquilinus*, were identified using the antennae of the worker termites by employing transcriptome screening, resulting in a large number of ORs, GRs, SNMPs, OBPs, and CSPs [49]. Another study recently published from Japan sequenced the genome of *R. speratus* and revealed 31 OBPs and 3 CSPs [50]. This study [50] also reported that the *R. speratus* genome (31 ORs, 25 GRs, 92 IRs, 5 SNMPs, and 10 CSPs) had been identified during its genome sequencing by [46]. To date, studies have been conducted on the trends of caste differentiation, including intercolonial aggression [51,52,53], and a study on the head transcriptome to analyze the expression of genes to understand caste differentiation and aggression is being conducted in *O. formosanus* [54]. Despite the extreme economic importance of *O. formosanus*, there has been no work conducted on the identification of chemosensory gene families in this species. The present study was conducted to identify the chemosensory gene families in *Odontotermes formosanus* workers to understand the molecular mechanism of the olfactory system of termites. First, we ran an RNA-seq analysis on the *Odontotermes formosanus* workers’ whole body. We then verified the gene results using gene-specific primers and performed qRT-PCR to understand their expression levels. We addressed the probable role of various olfactory genes in olfactory processing and how they are potentially involved in the social behavior (nestmate discrimination), gustation, host seeking, mate selection, and physiological functions of *O. formosanus* workers based on the data that we gathered.

## 2. Materials and Methods 

### 2.1. Sampling of Odontotermes formosanus

Three colonies were taken from the *Odontotermes formosanus* colonies maintained at the Nanning Institute of Termite Control in Nanning, Guangxi Province, China. Three biological replicates of ten healthy termite workers (the whole bodies of the workers) without any treatment were taken, promptly preserved in liquid nitrogen, and subsequently kept at −80 °C for sequencing. At this point, worker termite samples (whole-body, 10 healthy workers) were also obtained in three biological replicates with no prior treatment. These samples were quickly preserved in liquid nitrogen and then stored at −80 °C for the purpose of q RT-PCR validation conducted at later stages.

### 2.2. Isolation of Total RNA from Odontotermes formosanus Samples

According to the manufacturer’s instructions, total RNA was extracted utilizing an Invitrogen Life Technologies TRIzol Reagent (Invitrogen, Carlsbad, CA, USA). Total RNA concentration was determined using a NanoDrop-2000 (Thermo Scientific, Waltham, MA, USA). Additionally, an RNA Nano 6000 Assay Kit of the Bioanalyzer 2100 System (Agilent Technologies, Santa Clara, CA, USA) evaluated the RNA integrity.

### 2.3. Preparation of Library for Transcriptome Sequencing, De Novo Assembly, and Functional Annotation

After the total RNA of the sample was extracted, the mRNA was enriched with magnetic beads with Oligo (dT). The mRNA was divided into fragments using fragmentation buffer. The first cDNA strand was synthesized using an mRNA template with six-base random hexamers. Adding buffer, dNTPs, RNase H, and DNA polymerase I, a second cDNA strand was synthesized. The double-stranded cDNA was then end-repaired, poly (A) was added, sequencing was connected, magnetic beads were used for purification and fragment selection, and PCR amplification was performed to obtain the library. After the library was qualified, machine sequencing was carried out. 

#### 2.3.1. Bioinformatics Analysis

Clean data with high quality were obtained by filtering raw data, which removes adapter sequences and reads with low quality. By assembling clean data, putative genes were generated, which will be referred as unigenes. The quality of the library was then assessed via a randomness check and saturation analysis. With a qualified library, bioinformatics analyses were performed, including unigene expression quantification and gene structure analysis.

#### 2.3.2. Data Quality Control

The sequencing of the cDNA library was performed via MGI (MGI Tech Co., Ltd. Shenzhen, China) based on sequencing using synthesis technology on DNBSEQ-T7. This platform can produce a significant number of high-quality reads, known as raw reads, the majority of which can achieve Q30 or higher. Raw data were stored in FASTQ format. Each sample had two FASTQ files, containing cDNA reads measured at both ends.

We used fastp software (https://github.com/OpenGene/fastp; accessed on 1 December 2022) to implement the original data filtering principles. We removed reads containing adaptors, reads with an N ratio of more than 10%, reads with low quality (mass value less than 20), and reads with more than 50% base proportion. Then, we used FastQC (http://www.bioinformatics.babraham.ac.uk/projects/fastqc; accessed on 1 December 2022) software to carry out quality control on the clean data, and after the quality control was qualified, subsequent analysis was conducted. 

#### 2.3.3. Transcriptome Data Assembly 

The Trinity v2.5.1 program (http://trinityrnaseq.sourceforge.net/; accessed on 1 December 2022) [55] then processed the clean high-quality data obtained from the above steps for assembly. Then, the concatenated sequences were filtered and made de-redundant. The de-redundant sequences, namely the unigenes, were used as reference sequences for subsequent analysis. Using Trinity, reads were fragmented into smaller pieces, known as K-mers. These K-mers were then used as seeds to be extended into contigs, and then components were based on contig overlappings. Finally, De Bruijn was applied here to recognize transcripts in the components. The steps of the Trinity assembly were as follows: (1) Fragment reads into K-mers to generate a pool of K-mers. Erroneous K-mers were removed. (2) Choose K-mers with high frequency as seeds to extend into contigs in the manner of K-1 overlapping (K-mers with low complexity and rare K-mers were not chosen). Grow the contigs until the entire K-mer pool is exhausted. (3) Cluster the contigs obtained from step 2 into sets of connected components (the contigs that fulfill the following conditions were clustered into one of the following components: a. perfect overlap of k-1 bases; b. minimal number of reads spanning between two contig junctions; c. (k-1)/2 bases mapped back to both ends of (k-1)mer junction). (4) Construct a complete De Bruijn graph for each component. (5) Clarify the De Bruijn graph by merging nodes, pruning edges. (6) Report transcripts by tracing the actual reads in each De Bruijn graph. After obtaining the transcript sequence assembled by Trinity, in order to eliminate a false positive sequence, we compared the short reads after quality control to the transcript sequence assembled by Trinity, filtered out some of the assembled transcript sequences with coverage = 0, and then further clustered to eliminate redundancy. To further improve the elimination of redundancy, the filtered assembled transcripts were subjected to clustering with CD-HIT-EST, with a nucleotide identity threshold of 95%. The obtained transcripts were used as unigene sequences for subsequent analysis. 

After that, we annotated gene functions for unigenes. The gene-function annotation database for five databases includes NR-NCBI (non-redundant protein sequences) (http://blast.ncbi.nlm.nih.gov/Blast.cgi; accessed on 1 December 2022), [56], GO (Gene Ontology) [57], KEGG (Kyoto Encyclopedia of Genes and Genomes) [58], KOG (Eukaryotic Orthologous Groups of proteins) [59], and Swiss-Prot [60] databases. BLASTx was used to evaluate the *O. formosanus* transcripts included in the FASTA file that was acquired after transcriptome assembly against the nr database (NCBI non-redundant protein sequences, https://www.ncbi.nlm.nih.gov/; accessed on 1 December 2022). We utilized the BLAST2GO software (https://www.blast2go.com//; accessed on 1 December 2022). To locate sequences that were comparable to the query set; BLAST2GO takes advantage of the BLAST, which is the Basic Local Alignment Search Tool. BLAST2GO software was used to perform Gene Ontology (GO) annotation (http://www.geneontology.org; accessed on 1 December 2022), and BLAST hits were obtained. The genes were mapped to pathways using the web-based KEGG annotation server (http://www.genome.jp/tools/kaas/; accessed on 1 December 2022). The KOG database was mined using an online server available at http://www.ncbi.nlm.nih.gov/COG/; accessed on 1 December 2022. KOG (euKaryotic Orthologous Groups) is a eukaryotic version of COG, which contains orthologous information about proteins. In this case, a certain function can be annotated to all members of a single KOG ID by comparing all protein sequences on the genomes and applying the criterion of consistency of genome-specific best hits to define orthologous genes. The Swiss-Prot server web-based tool (available at http://www.expasy.ch/sprot; accessed on 1 December 2022) was used by BLASTx. 

### 2.4. Screening and Validation of Transcripts Encoding Putative Chemosensory Genes 

Candidate odorant binding proteins (OBPs), chemosensory genes (CSPs), odorant receptors (ORs), gustatory receptors (GRs), and sensory neuron membrane proteins (SNMPs) were determined using keyword searches and the outcomes of functional annotation. The NCBI (National Center for Biotechnology Information)’s BLASTx and tBLASTn programs were then used to independently verify each candidate unigene encoding OBPs, CSPs, ORs, GRs, and SNMPs at http://blast.ncbi.nlm.nih.gov/Blast.cgi (accessed on 1 July 2023). A program called ORF finder (http://www.ncbi.nlm.nih.gov/gorf/gorf.html; accessed on 1 July 2023) was used to identify the open reading frames (ORFs) of all chemosensory genes. It had been predicted from NCBI that conserved domains would be present in potential chemosensory genes (https://www.ncbi.nlm.nih.gov/Structure/cdd/wrpsb.cgi; accessed on 1 July 2023). The putative signal peptides were then identified by SignalP 4.1 (https://services.healthtech.dtu.dk/services/SignalP-4.1/; accessed on 1 July 2023). 

### 2.5. Sequence Analysis and Phylogenetic Tree Construction

Using NCBI-BLAST (http://blast.ncbi.nlm.nih.gov/; accessed on 1 July 2023), relevant homologous genes from other insect species that had similarities with each putatively discovered gene were found. The amino acid sequences of many potential chemosensory genes (OBPs, CSPs, ORs, GRs, and SNMPs) were systematically organized using ClustalW (https://www.ebi.ac.uk/Tools/msa/clustalo/; accessed on 1 July 2023). Multiple sequence alignments for protein sequences were performed to construct a phylogenetic tree using the ClustalW algorithm in MEGA 11 software. Utilizing MEGA 11, a phylogenetic tree was created employing the amino acid sequences from several insect species for OBPs, ORs, GRs, and SNMPs. Using bootstrap results from 1000 repetitions, the neighbor-joining approach was used with a Jones–Taylor–Thornton (JTT) model for the construction of a phylogenetic tree. The multiple sequence alignment for putative OBPs, ORs, GRs, and SNMPs was performed using DNAMAN software (version 5.2.2). The blast analyses, to compare putatively identified chemosensory genes for *Drosophila* genus, were analyzed using the blast tool on FlyBase (https://flybase.org/blast; accessed on 25 September 2023) using the annotated proteins (AA) database. 

### 2.6. Sequence Confirmation and qRT-PCR Validation

Using a Roche Real-time Light cycler 96 detection system (Mannheim, Baden-Wurttemberg, Germany), RT-qPCR was performed to evaluate the expression patterns of *OforCSP1*, *OforOBP1*, *OforOBP2*, *OforOR1*, *OforOR2*, *OforGR1*, *OforGR2*, *OforSNMP1* and *OforSNMP2* chemosensory genes. For the qRT-PCR study, gene-specific primers were developed based on sequences (Table S1). The samples for qRT-PCR include 2 × Syber Green PCR Master Mix (10 µL) of each gene-specific primer (0.5 L/10 M), cDNA (1 µL), and disinfected ultrapure water (8 µL), among other components (Aidlab, Beijing, China). Thermal cycling was carried out, beginning with a denaturation step at 95 °C for 3 min, then 40 cycles of 95 °C for 10 s and 55 °C for 30 s. The sensitivity of each primer set was confirmed by the melting curve, which displayed a single gene-specific peak, and the resulting linear standard curve was used to calculate the precision of amplification (E-value) using the formula E = 10^−1^/slope. A result of more than 90% was considered effective. To normalize the target gene expression and to address sample-to-sample variability, a reference gene, β-actin, was utilized [61]. The 2^−∆∆CT^ approach was used in order to perform ct-value quantification after each qPCR reaction had been carried out for each sample in three biological replicates with three technical replicates for each transcript.

### 2.7. Statistical Analysis

Data analysis was conducted using SPSS, Inc.’s computer program (Statistical Package for the Social Sciences, version 22.0 (SPSS, Inc., Chicago, IL, USA)). Tukey’s honest significant difference (HSD) test and analysis of variance were used to statistically analyze the qRT-PCR data. A statistically significant value was set at *p* < 0.05. 

## 3. Results

### 3.1. Overview of MGI Platform Sequencing and Unigene Assembly

The transcriptome of the worker caste of *Odontotermes formosanus* was generated using the sequencing system. Trinity processed the clean data with high quality obtained from the above steps for assembly. Then, the concatenated sequences were filtered and made de-redundant. The de-redundant sequences, namely unigenes, were used as reference sequences for subsequent analysis. Overall, 39,251,722 total reads were identified from the *O. formosanus* workers library, in which 39,036,281 were clean reads after removing the low-quality and adapter sequences. By further assembling the clean reads, 234,541 Trinity reads and 138,762 unigenes were extracted. The details of the unigenes and Trinity reads (total length, average length, etc.) are further explained in Table 1. The results of the blast analysis of putatively identified chemosensory genes in the *Drosophila* genus using the annotated proteins (AA) database are given in Table S2.

#### Functional Annotation

**Figure 1:**
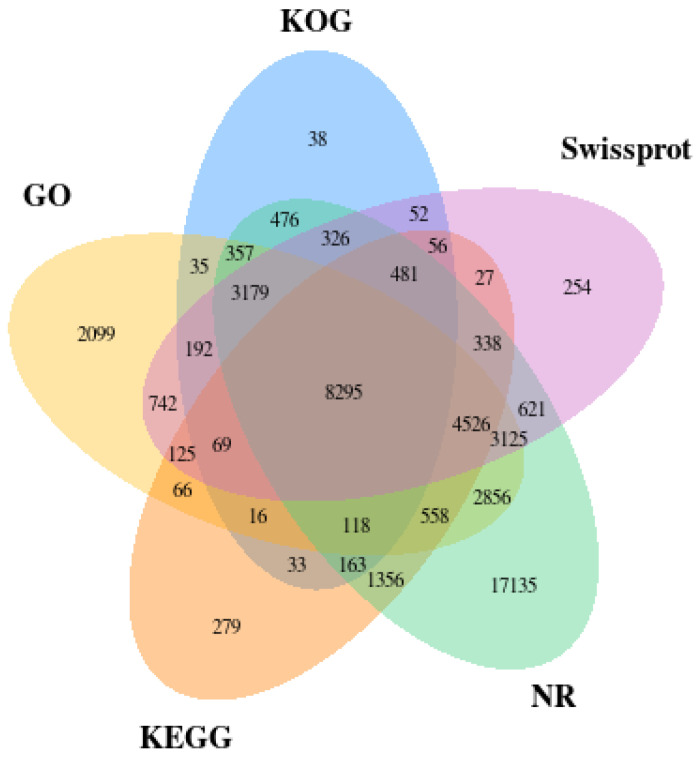
Functional annotations for the KOG, KEGG, NR, SwissProt, and GO databases are shown in a Venn diagram. The number of unigenes that share similarities across the datasets is shown by the overlapped areas between them.

The functional annotation is made for unigene-level sequences via homologous alignment. These transcripts from the same gene are compared with the same sequence in the database. Due to the redundancy of the various databases, some genes may be annotated in different databases simultaneously. We used five databases, including KOG, KEGG, NR, SwissProt, and GO, identified by the whole-body transcriptome of workers of *O. formosanus*. The results showed that 13,886 (10.01%), 16,506 (11.90%), 43,910 (31.64%), 22,408 (16.15%), and 26,358 (19.00%) unigenes were annotated in the KOG, KEGG, NR, SwissProt, and Go databases, respectively, comprising 47,993 (34.59%) unigenes overall among the total 138,762 unigenes found in the *O. formosanus* transcriptome assembly (Figure 1). 

The KOG (eukaryotic orthologous groups) database was used to annotate the unigenes. The KOG database contains orthologous information on proteins, for which 4852 classifications are available, and genes from the same orthologous classification share similar biological functions. So, a certain function has been annotated for all members of a single KOG ID. The genes with successful KOG annotation are classified according to the KOG group and are divided into 26 groups. In the KOG classification, the 3418 unigenes were classified as “general function prediction only”, the highest number of genes being classified in this group among the 26 groups of the KOG database, followed by the “signal transduction mechanisms”, comprising 1849 unigenes, and 1228 unigenes belonged to the “posttranslational modification, protein turnover, chaperones” group. The results are shown in Figure S1. 

**Figure 2:**
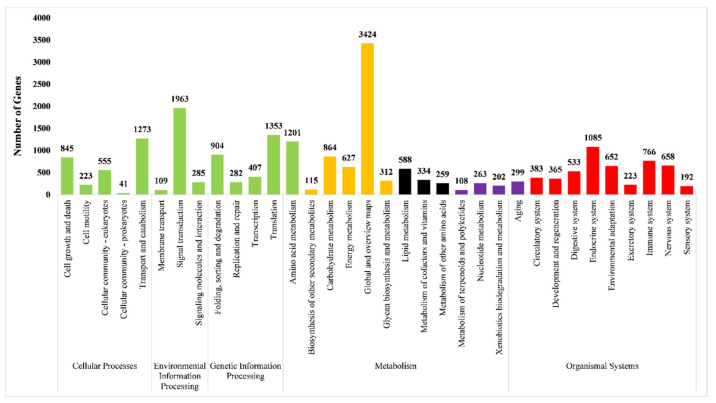
*O. formosanus* unigenes categorized according to KEGG (Kyoto Encyclopedia of Genes and Genomes) pathways.

The KEGG (Kyoto Encyclopedia of Genes and Genomes) is a systematic collection of gene functions within specific metabolic pathways. The KEGG was used to identify the pathway in cells among the unigenes identified in *O. formosanus*. In the KEGG pathway, the unigenes are divided into five categories, named cellular processes, environmental information processing, genetic information processing, metabolism, and organismal systems. In the cellular processes, a total of 1273 genes were classified as transport and catabolism. Signal transduction has the maximum number of 1963 genes in the environmental information processing category. In genetic information processing, a maximum of 1353 genes were mapped in the translation sub-category of cell function. In total, 3424 genes were mapped to the global and overview maps in the category of metabolism in KEGG. The endocrine system, in the category of organismal systems, has the highest number of genes, with 1085 in the KEGG pathway (Figure 2). 

**Figure 3:**
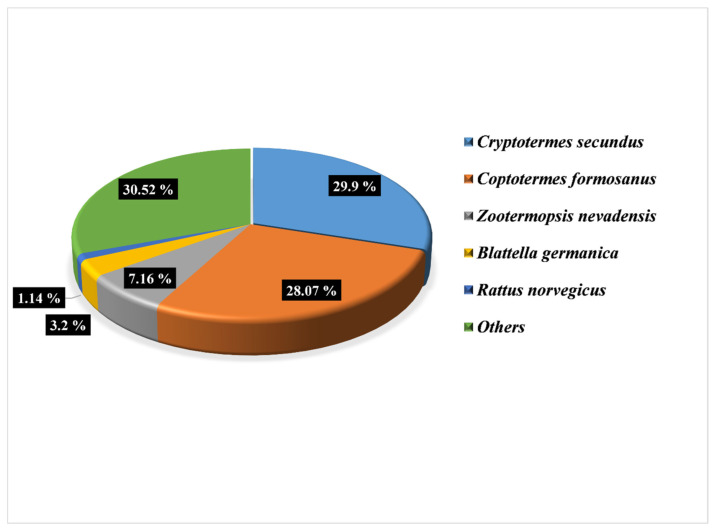
The distribution of species in the NR database, together with the greatest BLAST hit for each unigene, shown as a result of the query for similarity of unigenes to the NR database.

The NR (non-redundant protein database) contains the non-redundant protein sequences of all NCBI species. We used the BLAST function to align the unigene sequences to the NR protein sequences to obtain the NR annotation for each gene. Species information in NR annotation results showed that the annotated species (*Cryptotermes secundus*, *Coptotermes formosanus*, *Zootermopsis nevadensis*, *Blattella germanica*, *Rattus norvegicus*, and others) have considerable blast hits in the NR database, as shown in Figure 3. 

The function of genes was further explored by annotating the genes using the Gene Ontology (GO) from *O. formosanus*. Three main classifications—molecular function, cellular component, and biological process—are used to organize the entire database. In the GO classification, the percentage and number of unigenes are given in Figure S2. 

### 3.2. Identification of the Candidate Chemosensory Gene Families in O. formosanus

#### 3.2.1. Candidate OBPs and CSP

**Figure 4:**
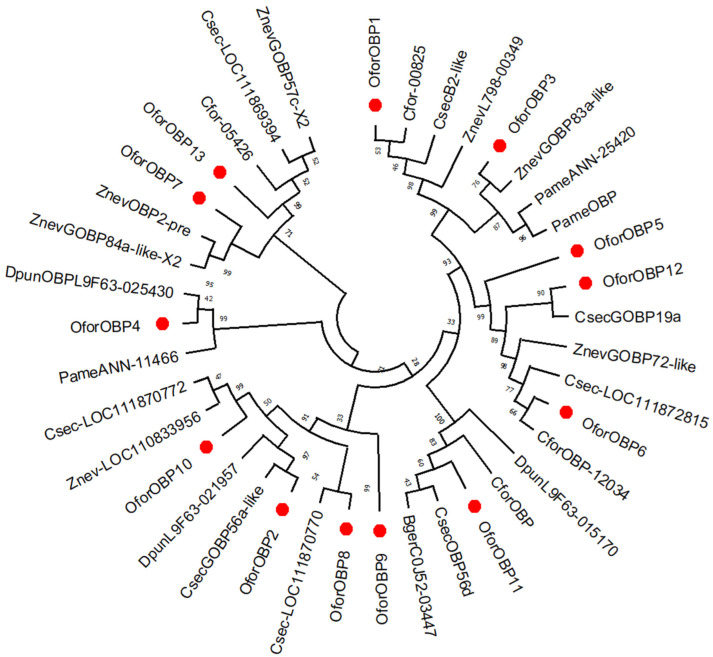
Phylogenetic tree of identified OBPs with the OBPs from different insect species. The identified OBPs are marked in red. The percentage of bootstrap is shown in black. The phylogenetic tree of 13 putative *OforOBPs* was constructed by using the sequences from the following insect species: *Cryptotermes secundus*, *Zootermopsis nevadensis* (*Csec*), *Coptotermes formosanus* (*Cfor*), *Periplaneta americana* (*Pame*), *Blattella germanica* (*Bger*), and *Diploptera punctate* (*Dpun*). GenBank accession numbers for all OBPs genes are: CsecB2-like; XP_023722114.1, ZnevL798-00349; KDR09912.1, Cfor-00825; GFG36297.1, CsecGOBP56a-like; XP_023719059.1, DpunL9F63-021957; KAJ9583696.1, ZnevGOBP83a-like; XP_021937238.1, PameANN-25420; KAJ4427767.1, PameOBP; ACI30685.1, DpunL9F63-025430; KAJ9576674.1, PameANN-11466; KAJ4441610.1, Csec-LOC111872815; XP_023722772.1, ZnevGOBP72-like; XP_021930874.1, Cfor-12034; GFG40302.1, ZnevGOBP84a-like_X2; XP_021924927.1, ZnevOBP2-pre; AAN15922.1, Csec-LOC111870770; XP_023719061.1, Znev-LOC110833956; XP_021928285.1, Csec-LOC111870772; XP_023719063.1, CsecGOBP56d; XP_023722057.1, CforOBP; AGM32399.1, BgerC0J52-03447; PSN48084.1, DpunL9F63-015170; KAJ9593295.1, CsecGOBP19a; XP_023722796.1, Cfor-05426; GFG37024.1, Csec-LOC111869394; XP_023716665.2, ZnevGOBP57c-X2; XP_021924930.1.

Our annotation results showed thirteen sequences from the transcriptome of *Odontotermes formosanus* workers, identified as odorant binding proteins (OBPs), which we named *OforOBP1*-*OforOBP13*. All of these 13 sequences contain start and stop codons, except *OforOBP13*. All the putative OBPs contain the conserved domain of the “PBP_GOBP super family” accession “c|11600” and match *Cryptotermes secundus*, *Zootermopsis nevadensis*, and *Coptotermes formosanus*, respectively, with different percentages of identity ranging from 40.65 to 86.21% (Table 2). The phylogenetic tree shows the various sub-groups among the identified OBPs and the OBPs of the other insect species (Figure 4). Multiple sequence alignment showed that there is 20.01% similarity among the newly identified putative OBPs of *O. formosanus* (Figure S3). One of the sequences was identified as the putative chemosensory protein CSP and named *OforCSP1*. *OforCSP1* has 52 amino acids with “OS-D super family” accession “c|04042” and has a 40.90% identity with *Plodia interpunctella.* Table 2 shows sequence features and best Blastp match results for *OforOBPs* and *OforCSP1*. 

#### 3.2.2. Candidate ORs

**Figure 5:**
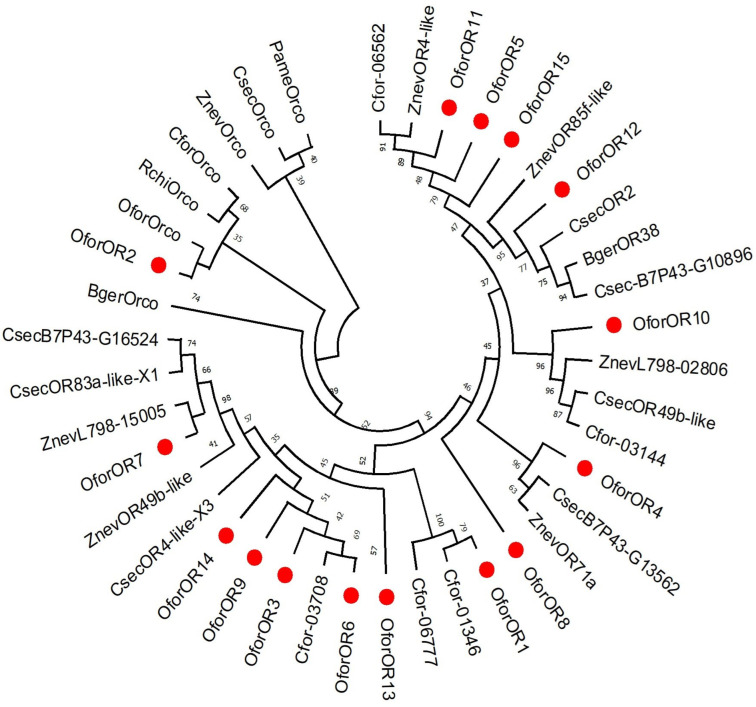
Phylogenetic tree of identified ORs with the ORs from different insect species. The identified ORs are marked red. The bootstrap percentages are shown in black. The phylogenetic tree of 15 putative *OforORs* was constructed by using the sequences from the following insect species: *Coptotermes formosanus*, *Odontotermes formosanus*, *Reticulitermes chinensis*, *Zootermopsis nevadensis*, *Cryptotermes secundus*, *Periplaneta americana*, and *Blattella germanica*. GenBank accession numbers for all ORs genes are: Cfor-01346; GFG35287.1, Cfor-06777; GFG31249.1, OforOrco; QZA87370.1, RchiOrco; QLJ82958.1, ZnevOrco; XP_021933609.1, CforOrco; XP_023716643.1, CsecOrco; XP_023716643.1, PameOrco; BDC30331.1, BgerOrco; PSN39983.1, CsecB7P43-G13562; PNF21445.1, ZnevOR71a; XP_021936751.1, Cfor-03708; GFG30955.1, ZnevL798-15005; KDR10322.1, CsecOR83a-like_ X1; XP_033609220.1, CsecB7P43-G16524; PNF24777.1, ZnevOR49b-like; XP_021936592.1, ZnevL798-02806; KDR21745.1, CsecOR49b-like; XP_033607259.1, Cfor-03144; GFG36204.1, Cfor-06562; GFG28618.1, ZnevOR4-like; XP_021923724.1, CsecOR2; XP_033607852.1, BgerOR38; PSN47015.1, ZnevOR85f-like; XP_021923636.1, CsecB7P43-G10896; PNF31354.1, CsecOR4-like-X3; XP_033606671.1.

In the current study, collectively, we have identified 15 odorant receptors (ORs) from *O. formosanus* workers’ whole-body transcriptome. The putative identified ORs were named *OforOR1-OforOR15*. All of these candidate odorant receptors have start and stop codons, except *OforOR6*, *OforOR13*, and *OforOR14*, and none of them contain signal peptides, as shown in Table 2. All of the putative *OforORs* contained the conserved domain of “7tm_6 super family” accession “c|20237”. *OforORs* match to *Coptotermes formosanus*, *Odontotermes formosanus*, *Zootermopsis nevadensis*, and *Cryptotermes secundus*, with the different percentages ranging from 51 to 100% in the Blastp match. Interestingly, *OforOR2* matches the previously identified odorant receptor coreceptor of *O. formosanus*. However, the *OforOR2* identified in our study had 346 amino acids while the previously identified Orco had 472 amino acids [62]. This shows that the N terminus is missing in our transcriptomic sequence. The multiple sequence alignment showed 18.65% similarity among all the putative identified *OforORs* (Figure S4). The phylogenetic analysis showed various distinctive groups, and each group is divided into subgroups, as shown in Figure 5. 

#### 3.2.3. Candidate GRs

In total, nine gustatory receptors (GRs) were identified from the annotation results. The putative identified GRs were named *OforGR1-OforGR9*. All of the putative identified *OforGR* sequences contain start and stop codons, except *OforGR4*, *OforGR6*, and *OforGR8;* among them, *OforGR3* contains the signal peptides, as shown in Table 2. The putative GRs contain the conserved domain of the “7tm_7 super family” accession “c|19976”. The putative *OforGRs* match *Cryptotermes secundus* and *Zootermopsis nevadensis*, with identity percentages varying from 60.29 to 81.25% in the Blastp match. The multiple sequence alignment showed 21.36% overall identity among the identified *OforGRs* (Figure S5). The phylogenetic analysis revealed various major groups among the putative OforGRs and GRs from the other insect species (Figure S6).

#### 3.2.4. Candidate SNMPs

Four putative sensory neuron membrane proteins (SNMPs) were identified in the annotation results. The putative SNMPs were identified as *OforSMMP1*-*OforSNMP4.* Two SNMPs sequences contain start and stop codons, while *OforSNMP3* and *OforSNMP4* have incomplete ORFs. The candidate *OforSNMPs* contain the conserved domain “CD36 super family” accession “c|10574”. The *OforSNMPs Zootermopsis nevadensis*, *Coptotermes formosanus*, and *Blattella germanica* have identities ranging from 49.14 to 89.11% in the results of the Blastp match (Table 2). The overall similarity of the putative *OforSNMPs* is 33.62% in the results of multiple sequence alignment (Figure S7). The phylogenetic analysis showed multiple distinctive groups with the different insect species (Figure S8).

### 3.3. Expression Patterns of the Putative Genes from the Chemosensory Families

**Figure 6:**
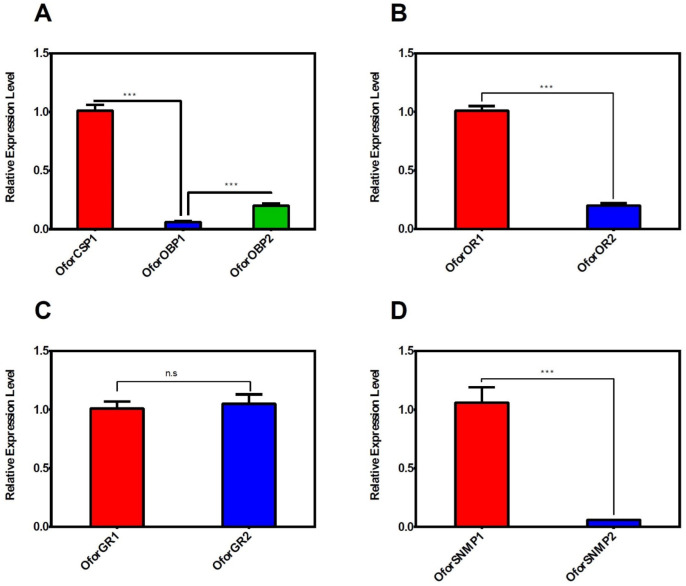
The relative level of mRNA expression of each putative CSP, OBP (**A**), OR (**B**), GR (**C**), and SNMP (**D**) in *O. formosanus* workers’ whole body. Fold variations of *OforOBPs* are relative to the *OforCSP1* transcripts of workers *O. formosanus*. Fold variations of OforOR2, OforGR2, and OforSMPP2 are relative to the *OforOR1*, *OforGR1*, and *OforSNMP1* transcript of workers *O. formosanus*. The number of stars reveals the difference in *p* values at a level of significance, i.e., *** *p* ≤ 0.001, while “n.s” denotes non-significant differences.

The qRT-PCR was performed for nine putative chemosensory genes identified in *O. formosanus*: *OforCSP1*, *OforOBP1*, *OforOBP2*, *OforOR1*, *OforOR2*, *OforGR1*, *OforGR2*, *OforSNMP1*, and *OforSNMP2*. qRT-PCR was performed using the whole bodies of *O. formosanus* workers. The mRNA expression was higher among the putative identified chemosensory genes. The expression of *OforCSP1* and *OforOBP2* was significantly higher than that of *OforOBP1*. *OforOR1* had a significantly higher expression compared with *OforOR2*. The mRNA expressions of *OforGR1* and *OforGR2* did not show statistically significant results. A significantly higher expression was also observed in *OforSNMP1* as compared with *OforSNMP2*, as shown in Figure 6. 

## 4. Discussion

*Odontotermes formosanus* (Shiraki) is a most destructive pest, causing substantial economic consequences for agricultural crops as well as infrastructure. Numerous previous studies have proven the importance of olfactory behavior modulation technologies in pest management [30]. How the reception of odorant molecules in antennae by binding proteins activates signal transduction remains unclear [50]. Chemical signals control the social structure of termites; however, the role of chemosensory genes during chemical communication and the way in which these cues are recognized by other individuals are unknown. In this study, we have identified various chemosensory genes in the *O. formosanus* transcriptome. Moreover, we have drawn on a phylogenetic analysis among termites and some other insect species and their expression patterns to uncover their possible roles in termite chemoreception. 

In this study, we identified 42 chemosensory genes, including 13 OBPs, 1 CSP, 15 ORs, 9 GRs, and 4 SNMPs, using RNA-seq analysis of the worker caste of *O. formosanus*. All of the putatively identified genes have not been previously reported in *O. formosanus*, except *OforOR2* [62]. Our results are based on de novo assembly data obtained via RNA-seq and comparable to *Reticulitermes speratus*, in which 31 OBPs identified and *Z. nevadensis* with 29 OBPs identified [42,50]. There are 52 OBP genes in *D. melanogaster* [63]; however, the highest number of 109 OBPs was observed in *B. germanica*, showing the variability of the number of OBPs among social insects [64]. OBPs play a role in smell perception in the insect olfactory system by participating in physiological sensitivity [65,66,67,68,69]. The 10–20 kDa, generally a small molecular size for N terminus signal peptides, are the ordinary traits of OBPs in insects [63,70,71,72], and this is reflected in our findings. OBPs and CSPs are plentiful in antennae [73], where they are responsible for olfaction and have a role in survival and reproduction [74]. Similarly, in our study, only one (*OforCSP1*) was identified. In the antennae of *D. melanogaster*, the first CSP gene was discovered, and four CSP genes have been identified in *Drosophila melanogaster* so far. These proteins were given the names olfactory specific protein D (OS-D) and pheromone-binding protein A-10 (A-10) because of their predominant expressions in the antennae [75,76]. The DmelCSP3 gene is hypothesized to have a role in the regeneration and development of tissues in *D. melanogaster* and has been identified as a potential target in the context of embryonic and tissue development. Also, *DmelCSP2* has broad functionality in the process of tissue remodeling after damage or during developmental stages, as well as its significant upregulation at metamorphosis and in responses to viral and bacterial stimuli [77]. The CSP genes from 11 fig wasps have a close genetic relationship to the DmelCSP1 and DmelCSP2 genes. These genes may possess comparable functions to DmelCSP1 and DmelCSP2 in fig wasps [78]. In termites, 3 CSPs in *R. speratus* [48], 6 CSPs in *R. aculabialis* [74], 10 CSPs in *N. cubanus*, 6 CSPs in *P. simplex*, and 9 CSPs in *I. inquilinus* [49] were identified, which shows a higher number of CSPs [49] than the prior study of genome annotation in *Z. nevadensis* and *C. secundus* [47]. Even though chemosensory genes are dispersed to every body part in insect, they are mostly found in chemosensory organs, i.e., antennae and maxillary palps, and CSPs are distributed to every important organ of the insect body. CSPs are found in the ejaculatory bulb of *D. melanogaster*, wings of *L. migratoria*, in the proboscis of *M. brassicae*, in the tarsi of *S. gregaria*, in the female moth’s labial palps (*C. cactorum*), in the brain of the honey bee *Apis mellifera*, and in the reproductive system of *Helicoverpa* spp., as well as in the legs of some insects [22,76,79,80,81,82,83]. CSPs are expressed in the sex pheromone gland of the cabbage armyworm *Mamestra brassicae*, suggesting a function in the carrying and discharge of pheromones [84]. In another study, the chemosensory genes from six different organs of the *Spodoptera exigua* identified 159 putative chemosensory genes from transcriptomic data and found that 28 GRs were found in gustatory organs, but not in olfactory organs [85]. A high number of chemosensory genes is always present in the chemosensory organs; however, the transcriptome and relative expression data show that these genes are also present in other insect organs and spread to other body parts, including legs, wings, abdomens, heads, antennae, and the whole body in lower numbers. This supports the hypothesis that these genes are involved in developmental and physiological processes and act as chemical carriers.

Similarly, among insects, aphids need to discriminate across numerous host plant species, which boosts olfactory recognition performance. Thus, as aphid evolution becomes more sophisticated, olfactory recognition-related genes develop in quantity, structure, and function. Contrary to this, in *M. sanborni*, the host plant range is limited to *Chrysanthemum* cultivars; therefore, the number of chemosensory genes may be lower than in aphids [86]. This phenomenon might be similar in termites. According to the results of the phylogenetic sequence analysis, even though, in our results, some proteins were highly similar to other termite proteins because of their high sequence homology, the homology between other insect species is very low, and only a small number of sequences from other insect species have been identified in the Blastp search. This phenomenon demonstrates termite species conservation.

Turning now to *OforORs*, we identified 15 ORs in *O. formosanus*. Comparable to *D. melanogaster*, 60 genes from the OR family encode 62 receptors [87], whereas in some ant species, this number ranges up to ~400 [28,88,89]. Similarly, a large number of ORs has been identified from the transcriptomes of three species of termites: 50 ORs in *P. simplex*, 30 ORs in *N. cubanus*, and 28 ORs in *I. inquilinus* [49]. The genomes of *C. secundus* and *Z. nevadensis* display 42 and 69 ORs, respectively [42,47], and in *R. speratus*, the number is 22 ORs or less [48]. It has been shown that Orco binds to ligand-specific ORs to make heterodimeric complexes, which are needed for OR trafficking [17,90]. Orco has remarkable levels of conservation across all insect species [91,92]. Among Blattodeans, *B. germanica* had the most ORs (134), which might be because it has a big genome, chromosomal translocations, and a greater proportion of gene family expansions [47]. In Isoptera, ORs of the distinct termite species show a greater conservation for the very diverse OR family [49]. ORs typically include less than 500 amino acids [93]. ORs have less homology across various insects and are scattered among related species, which is possibly connected to the involvement of ORs in odorant identification [94]. Previously, RNAi of *Orco* and *5-HTT* genes were suggested to regulate nestmate discrimination in *O. formosanus*, according to the findings of one study [95]. In another study, silencing an *Orco* in *R. chinensis* and *O. formosanus* decreased their ability to perceive trail pheromones and their distance and velocity in both species while increasing the angular velocity in *R. chinensis* [62]. 

The annotation results showed nine GRs from transcriptome assembly. There are 60 genes from the GR family encoding 68 receptors in *D. melanogaster* [87,96,97], while the largest known family of GRs in insects is from *B. germanica*, which has 431 genes potentially encoding 545 proteins [64]. Comparable to *R. speratus*, in which 7 GRs were previously found [48], 20 GRs in *N. cubanus*, 25 GRs in *P. simplex*, and 26 GRs in *I. inquilinus* were found [49]. Apart from the other olfactory receptors, sugar receptors, bitter receptors, and pheromone recognition receptors are all examples of gustatory receptors that are expressed in olfactory organs and have the potential to play a role in insect sensory perception [98]. 

SNMPs (sensory neuron membrane proteins) are found in the antennae [99]. Our results annotated four transcripts as SNMPs. In *D. melanogaster*, two SNMPs have been identified and named as DmelSNMP1 and DmelSNMP2. SNMP genes are mostly found in the antennae of insects [99,100]. However, in *Aedes aegypti* and in *D. melanogaster*, they are also expressed in the non-olfactory regions of the wings and legs [101,102]. Previously, six genes from *P. simplex* and *I. inquilinus* and five from *N. cubanus* were identified. The first SNMPs were discovered in *Antheraea polyphemus*. SNMPs are the least frequent of the six main chemosensory gene families in aphids, with six being found in *M. persicae*. According to previous studies, SNMPs, which are transmembrane proteins that are a part of the highly conserved cluster of the differentiation 36 (CD36) family, are involved in the detection of lipid-derived pheromones in insects [103,104]. The sole CD36 family protein that appears in neurons is SNMP. SNMPs feature an extracellular ring and a transmembrane domain at both their C- and N-termini. MsanSNMP1 is a classic example of the aforementioned traits [86]. 

Analyzing the patterns of chemosensory gene expression is a useful method for determining how genes work. The expression of various chemosensory genes has been found in all parts of the insect body, including the antennae, head, thorax, legs, and abdomen. The expression patterns in eusocial insects change in the castes (soldiers, workers, alates), as well as in different tissues. Interestingly, studies also support that some chemosensory genes showed significantly higher expression of some genes in castes, as well as in different tissues [48,50,95]. In addition, chemosensory organs (antennae, maxillary palps, etc.) are major insect parts in which the chemosensory genes are predominantly expressed, mostly for OBPs and CSPs, and for the other genes including IRs, ORs, GRs, and SNMPs. Comparing the expression patterns of the chemosensory genes from the worker termites in our study with previously published data on *M. separata*, *MsCSP5* has been predominantly expressed in adults, particularly in female antennae. MsCSP8 in *M. separata* was also expressed in adults, predominantly in the antennae, legs, and wings [105,106]. NlugCSP10 was highly expressed throughout phases of life of *Nilaparvata lugens* [107]. The expression patterns of OBPs and CSPs in *S. avenae*, *M. sanborni*, *M. persicae*, and *Schlechtendalia chinensis* are the same as in aphids [69,86,108,109,110]. In termites, silencing *Orco* caused general olfaction deficiencies and impaired the detection of trail pheromones in termites [95]. 

In addition to the odorant receptors (ORs), gustatory receptors (GRs), and ionotropic receptors (IRs), pickpocket (PPK) receptors, otopetrin-like proteins (Otop), transient receptor potential (TRP) channels, and opsin proteins are also present in insects. Mechano- and chemo-sensing are only two of the numerous tasks that are mediated by insect pickpocket (PPK) receptors [111]. PPKs were discovered in 26 species across eight orders, with 578 genes spread across seven subfamilies. There are a total of 31 PPKs in the *Drosophila melanogaster* genome, spread over seven different PPK subfamilies [112]. The otopetrin-like protein (Otop) family is a different class of receptors that have been tailored to the acidic taste. Mammals and insects share conservation in the Otop family [113]. OtopLA, OtopLB, and OtopLC are the three otopetrin-like proteins that are encoded by *Drosophila*. These proteins have a modest amount of amino acid homology with the OTOP1 proteins found in mice and humans [114,115]. Cation channels that belong to the type known as transient receptor potential (TRP) channels are also engaged in sensory signaling processes like pain, vision, taste, and touch [116]. In addition to these receptors, the structure of opsin proteins is a primary factor in determining the degree to which visual pigments are sensitive to varying wavelengths of light. Opsin proteins are essential components of insect vision [117]. In *D. melanogaster*, seven proteins are characterized as opsins that respond to blue (*SWS*), green (*LWS*), and UV light [118,119,120]. Although, in the current study, we have identified OBPs, CSPs, ORs, GRs, and SNMPs from the transcriptome of *O. formosanus*, the chemosensation in insects is not limited to these gene families, and there is still scope to identify PPKs, Otops, TRP channels, and opsin proteins from termites that are understudied and require further research. Nonetheless, beyond chemoreception, chemosensory genes serve an important role in regulating social behavior (nestmate discrimination), gustation, host seeking, mate selection, and physiological functions by facilitating regeneration and development, transporting visual pigments, and even providing insecticide resistance in insects. The high expression of *OforCSP1*, *OforOBP2*, *OforOR1*, and *OforSNMP1* suggests that further functional studies on the chemosensory genes in termites might disclose their role in chemoreception, even though future studies are needed.

## 5. Conclusions

Forty-two candidate genes were identified as the chemosensory genes for the first time in *O. formosanus* by using the RNA-seq data of the worker caste. In addition, a large number of unigenes were also identified for future research. Furthermore, we performed expression analysis via qRT-PCR. The high levels of gene expression show that these genes might be involved in chemoreception, and beyond chemoreception, they might be functional in physiological processes and social behavior, including caste differentiation, nestmate and non-nestmate discrimination, and the performance of colony functions among members, such as caste, age, and age-specificity. Our results provide new insights into the chemosensory genomics needed to perform colony functions and the evolution of polyphenism in termites. The molecular underpinnings of the functional olfactory system in termites provide a new direction for understanding chemical communication among termites as well as semiochemical-based termite pest management in the future.

## Figures and Tables

**Table 1:** Summary of de novo assembly results of *Odontotermes formosanus*.

Parameters	*Odontotermes formosanus*
Total reads	39,251,722
Clean reads	39,036,281
Total bases	5,927,010,022
Clean bases	5,872,123,432
Q20 (%)	98.08
Q30 (%)	94.14
GC (%)	41.27
Total number of unigenes	138,762
Total length of unigenes	121,676,404
Average length of unigenes	876.87
Median length of unigenes	431
Maximum length of unigenes	27,662
Minimum length of unigenes	201
Total number of Trinity reads	234,541
Total length of Trinity reads	311,356,994
Average length of Trinity reads	1327.52
Median length of Trinity reads	588
Maximum length of Trinity reads	27,662
Minimum length of Trinity reads	185

Note: Q20 (%): The aggregate number of bases with precision identification reaches 99.0%. Q30 (%): The aggregate number of bases with precision identification reaches 99.9%.

**Table 2:** The identified chemosensory genes of *Odontotermes formosanus* and the results of Blastp match.

Gene Name	GenBank Accession Number	Length (aa)	Signal Peptide	Sequence * (Yes/No)	Domain Incomplete **	Conserved Domain	Blastp Match					
							Species	Accession Number	Score	QC (%)	E-Value	Identity (%)
*OforOBP1*	OR651388	106	1-18	Yes	C	PBP_GOBP super family	*Cryptotermes secundus*	XP_023722114.1	118	80	2 × 10^−311^	64.71
*OforOBP2*	OR651389	155	1-23	Yes	-	PBP_GOBP super family	*Cryptotermes secundus*	XP_023719059.1	194	98	1 × 10^−60^	59.87
*OforOBP3*	OR651390	139	1-18	Yes	-	PBP_GOBP super family	*Zootermopsis nevadensis*	XP_021937238.1	234	100	2 × 10^−76^	79.14
*OforOBP4*	OR651391	138	1-19	Yes	-	PBP_GOBP super family	*Cryptotermes secundus*	XP_023725124.1	201	99	1 × 10^−63^	64.96
*OforOBP5*	OR651392	146	1-24	Yes	-	PBP_GOBP super family	*Cryptotermes secundus*	XP_023722798.1	175	84	4 × 10^−53^	63.41
*OforOBP6*	OR651393	72	-	Yes	C	PBP_GOBP super family	*Cryptotermes secundus*	XP_023722772.1	123	100	3 × 10^−32^	79.17
*OforOBP7*	OR651394	160	1-21	Yes	-	PBP_GOBP super family	*Zootermopsis nevadensis*	XP_021924927.1	248	100	2 × 10^−81^	75
*OforOBP8*	OR651395	170	1-20	Yes	-	PBP_GOBP super family	*Cryptotermes secundus*	XP_023719061.1	190	82	2 × 10^−58^	59.57
*OforOBP9*	OR651396	210	1-19	Yes	-	PBP_GOBP super family	*Cryptotermes secundus*	XP_023704937.1	176	100	1 × 10^−51^	40.65
*OforOBP10*	OR651397	144	1-20	Yes	-	PBP_GOBP super family	*Zootermopsis nevadensis*	XP_021928285.1	167	96	3 × 10^−50^	51.80
*OforOBP11*	OR651398	151	1-24	Yes	-	PBP_GOBP super family	*Zootermopsis nevadensis*	XP_021937236.1	272	100	3 × 10^−91^	82.78
*OforOBP12*	OR651399	146	1-24	Yes	-	PBP_GOBP super family	*Cryptotermes secundus*	XP_023722796.1	193	99	4 × 10^−60^	62.07
*OforOBP13*	OR651400	58	-	NO	NC	PBP_GOBP super family	*Coptotermes formosanus*	GFG37024.1	107	100	4 × 10^−28^	86.21
*OforCSP1*	OR651283	52	-	Yes	N	OS-D super family	*Plodia interpunctella*	XP_053615073.1	52	94	3 × 10^−06^	44.90
*OforOR1*	OR651429	307	-	Yes	NC	7tm_6 super family	*Coptotermes formosanus*	GFG35287.1	515	99	4 × 10^−179^	80.59
*OforOR2*	OR651430	346	-	Yes	N	7tm_6 super family	*Odontotermes formosanus*	QZA87370.1	718	100	0.00	100
*OforOR3*	OR651431	417	-	Yes	-	7tm_6 super family	*Zootermopsis nevadensis*	KDR19176.1	479	96	2 × 10^−164^	57.32
*OforOR4*	OR651432	111	-	Yes	N	7tm_6 super family	*Cryptotermes secundus*	PNF21445.1	141	80	1 × 10^−39^	74.16
*OforOR5*	OR651433	479	-	Yes	-	7tm_6 super family	*Coptotermes formosanus*	GFG30512.1	605	99	0.0	59.41
*OforOR6*	OR651434	413	-	NO	-	7tm_6 super family	*Zootermopsis nevadensis*	KDR19177.1	508	95	7 × 10^−176^	59.90
*OforOR7*	OR651435	286	-	Yes	N	7tm_6 super family	*Zootermopsis nevadensis*	KDR10322.1	485	99	4 × 10^−169^	80.35
*OforOR8*	OR651436	110	-	Yes	N	7tm_6 super family	*Zootermopsis nevadensis*	XP_021932574.1	109	88	5 × 10^−28^	51
*OforOR9*	OR651437	275	-	Yes	N	7tm_6 super family	*Coptotermes formosanus*	GFG34088.1	390	91	1 × 10^−133^	71.31
*OforOR10*	OR651438	174	-	Yes	N	7tm_6 super family	*Zootermopsis nevadensis*	XP_021915284.1	243	98	5 × 10^−76^	68.60
*OforOR11*	OR651439	121	-	Yes	N	7tm_6 super family	*Coptotermes formosanus*	GFG28618.1	192	85	3 × 10^−56^	83.65
*OforOR12*	OR651440	124	-	Yes	N	7tm_6 super family	*Cryptotermes secundus*	XP_033607852.1	217	100	1 × 10^−69^	81.45
*OforOR13*	OR651441	96	-	NO	N	7tm_6 super family	*Cryptotermes secundus*	XP_033606671.1	112	100	2 × 10^−28^	54.17
*OforOR14*	OR651442	80	-	NO	N	7tm_6 super family	*Coptotermes formosanus*	GFG41080.1	129	100	1 × 10^−35^	73.75
*OforOR15*	OR651443	157	-	Yes	N	7tm_6 super family	*Coptotermes formosanus*	GFG30511.1	218	89	1 × 10^−51^	62.86
*OforGR1*	OR651376	273	-	Yes	N	7tm_7 super family	*Cryptotermes secundus*	PNF40292.1	404	87	6 × 10^−137^	81.25
*OforGR2*	OR651377	290	-	Yes	N	7tm_7 super family	*Cryptotermes secundus*	XP_023711366.1	447	100	6 × 10^−154^	73.79
*OforGR3*	OR651378	308	1-20	Yes	N	7tm_7 super family	*Cryptotermes secundus*	XP_023708030.1	460	100	1 × 10^−158^	70.78
*OforGR4*	OR651379	348	-	NO	-	7tm_7 super family	*Cryptotermes secundus*	XP_023704213.2	492	99	1 × 10^−170^	69.71
*OforGR5*	OR651380	248	-	Yes	N	7tm_7 super family	*Zootermopsis nevadensis*	XP_021929494.1	368	95	2 × 10^−123^	74.15
*OforGR6*	OR651381	116	-	NO	N	7tm_7 super family	*Zootermopsis nevadensis*	XP_021920101.1	138	87	7 × 10^−39^	66.67
*OforGR7*	OR651382	82	-	Yes	N	7tm_7 super family	*Cryptotermes secundus*	PNF27532.1	112	98	7 × 10^−28^	67.90
*OforGR8*	OR651383	117	-	NO	C	7tm_7 super family	*Cryptotermes secundus*	XP_023708030.1	80.5	56	1 × 10^−14^	60.29
*OforGR9*	OR651384	59	-	Yes	N	7tm_7 super family	*Zootermopsis nevadensis*	XP_021915038.1	79.7	96	1 × 10^−15^	68.42
*OforSNMP1*	OR651358	496	-	Yes	-	CD36 super family	*Zootermopsis nevadensis*	XP_021913553.1	1461	100	0.00	89.11
*OforSNMP2*	OR651359	515	-	Yes	-	CD36 super family	*Zootermopsis nevadensis*	XP_021913531.1	921	100	0.00	83.50
*OforSNMP3*	OR651360	190	-	NO	C	CD36 super family	*Coptotermes formosanus*	GFG35002.1	295	99	2 × 10^−94^	72.49
*OforSNMP4*	OR651361	118	-	NO	N	CD36 super family	*Blattella germanica*	AMA98193.1	133	98	7 × 10^−36^	49.14

**Sequence** *: presence of start and stop codons at both termini in transcript sequence (Yes/No); Domain Incomplete **: if the hit to a conserved domain results is one of the following: **N**: incomplete at the N-terminus; **C**: incomplete at the C-terminus; **NC**: incomplete at both the N-terminus and C-terminus; **(-)**: the hit to a conserved domain is complete and is expressed as a dash (-).

## Data Availability

The authors confirm that the data supporting the findings of this study are available in the Supplementary Materials. The sequencing data generated from this project were submitted to the NCBI SRA database linked to the BioProject PRJNA1021793.

## Supplementary Materials

The following supporting information can be downloaded at: https://www.mdpi.com/article/10.3390/insects14110883/s1. Table S1. The primers used in the RT-PCR and qRT-PCR for identification of putative chemosensory genes in *O. formosanus*; Table S2. The Blastp match of *O. formosanus* of identified chemosensory protein genes with *Drosophila* (genus) on FlyBase blast tool using annotated proteins (AA) database; Figure S1. KOG annotation of unigenes in the *O. formosanus* workers; Figure S2. Gene function categorization of *O. formosanus* worker unigenes using the Gene Ontology (GO) system; Figure S3. The multiple sequence alignment of *O. formosanus* putative OBPs. There is a 20.01% amino acid similarity between all the putative OBPs; Figure S4. The multiple sequence alignment of *O. formosanus* putative ORs. There is a 18.65% amino acid similarity between all the putative ORs; Figure S5. The multiple sequence alignment of *O. formosanus* putative GRs. There is a 21.36% amino acid similarity between all the putative GRs; Figure S6. Phylogenetic tree of identified GRs with the GRs from different insect species. The identified GRs are marked red; Figure S7. The multiple sequence alignment of *O. formosanus* putative SNMPs. There is a 33.62% amino acid similarity between all the putative SNMPs; Figure S8. Phylogenetic tree of identified SNMPs with the SNMPs from different insect species. The identified SNMPs are marked red.; Gene Sequences; Chemosensory Genes Identified in the Transcriptome assembly of *O. formosanus*.
