# Supplementary material for: Uncovering the Chemosensory System of a Subterranean Termite, Odontotermes formosanus (Shiraki) (Isoptera: Termitidae): Revealing the Chemosensory Genes and Gene Expression Patterns
Source: Insects. 2023 Nov 15;14(11):883. doi: 10.3390/insects14110883 (PMC10672159; doi:10.3390/insects14110883)
Supplement: Supplementary file 1 [file insects-14-00883-s001.zip › insects-2617786-supplementary.pdf]

**Table S1 The primers used in the RT-PCR and qRT-PCR for identification of putative chemosensory genes in *O. formosanus***

| Primer (For qRT-PCR) | Primer sequences (5´to 3´) |
|---|---|
| *OforCSP1*-F | TGCAGCAAATGTTCTCCTAGC |
| *OforCSP1*-R | GCTTTCTTCCCGTCAGGATCG |
| *OforOBP1*-F | AAGGCGATGATGAAAATGCTGC |
| *OforOBP1*-R | GCCTTTCATGGCTCCTAGAAT |
| *OforOBP2*-F | TTACTTCTATTGATTGTTGTGGC |
| *OforOBP2*-R | TTCCTCCTGGCACTTTGCTTG |
| *OforOR1*-F | TTCAATTCAATCTTCACAGGCTACG |
| *OforOR1*-R | TAAATTTGCTAGGCGTTCTAAGTCAG |
| *OforOR2*-F | ATGCTACGTTCGTGGTATCCC |
| *OforOR2*-F | AGCCAGAAATAGCCACAGGAT |
| *OforGR1*-F | GACAGAGTGGCACTATCAGGAATG |
| *OforGR1*-R | GCAGAAAGGGACACCGAAACTA |
| *OforGR2*-F | ACATCTTACGGTTATCCAAGGAA |
| *OforGR2*-R | GCCATGAGGTGTTCGGCTGT |
| *OforSNMP1*-F | GGTTTCCCGTCTCTAATCCG |
| *OforSNMP1*-R | GTCCATTGGATTTGTTATATTGAA |
| OforSNMP2-F | GGGATGACCATCTTCGTGCTA |
| *OforSNMP2*-R | CCAAATTTCCCTTCTCTCAGC |
| **Actin gene** | |
| *β-Actin*-F | GGTCTCTTATCTGCTCTATCA |
| *β-Actin*-R | TCTGCTATACTTCCTTCCTG |

**Table S2.** The Blastp match of *O. formosanus* of identified chemosensory protein genes with *Drosophila* (genus) on FlyBase BLAST tool using annotated proteins (AA) database

| Gene Name | ORF (aa) | FlyBase Blastp Match | | | | | | |
|---|---|---|---|---|---|---|---|---|
| | | Description | Species | ID | Score | E-value | Identities (%) | Positives (%) |
| *OforOBP1* | 106 | Obp83a-PB | *Drosophila melanogaster (Dmel)* | FBpp0311427 | 40.0466 | 0.000414628 | 37.5 | 54.2 |
| *OforOBP2* | 155 | Obp56d-PB | *Drosophila melanogaster (Dmel)* | FBpp0311727 | 44.2838 | $4.68988e^{-05}$ | 23.8 | 42.7 |
| *OforOBP3* | 139 | Obp83a-PB | *Drosophila melanogaster (Dmel)* | FBpp0311427 | 68.5514 | 1.99131e-12 | 28 | 54.2 |
| *OforOBP4* | 138 | Obp28a-PA | *Drosophila melanogaster (Dmel)* | FBpp0076996 | 47.7506 | 3.49948e-06 | 39 | 59.3 |
| *OforOBP5* | 146 | Obp19a-PC | *Drosophila melanogaster (Dmel)* | FBpp0297995 | 63.5438 | 5.9664e-11 | 29 | 55.9 |
| *OforOBP6* | 72 | Obp19a-PC | *Drosophila melanogaster (Dmel)* | FBpp0297995 | 35.8094 | 0.00851655 | 26.2 | 53.8 |
| *OforOBP7* | 160 | Obp56g-PB | *Drosophila melanogaster (Dmel)* | FBpp0089127 | 42.3578 | 0.000206394 | 31.7 | 52.5 |
| *OforOBP8* | 170 | Obp56e-PB | *Drosophila melanogaster (Dmel)* | FBpp0309474 | 40.4318 | 0.000806394 | 26.9 | 46.3 |
| *OforOBP9* | 210 | CG17994-PA | *Drosophila melanogaster (Dmel)* | FBpp0085345 | 31.9574 | 0.431517 | 39.6 | 54.2 |
| *OforOBP10* | 144 | Obp56c-PB | *Drosophila melanogaster (Dmel)* | FBpp0089321 | 37.7354 | 0.00371322 | 23.5 | 40.9 |
| *OforOBP11* | 151 | lush-PB | *Drosophila melanogaster (Dmel)* | FBpp0290704 | 55.0694 | 2.86563e-08 | 37.8 | 59.5 |
| *OforOBP12* | 146 | Obp19a-PC | *Drosophila melanogaster (Dmel)* | FBpp0297995 | 63.5438 | 6.64997e-11 | 27.4 | 47.3 |
| *OforOBP13* | 58 | Obp99b-PB | *Drosophila melanogaster (Dmel)* | FBpp0305400 | 26.9498 | 4.33555 | 31 | 58.6 |
| *OforCSP1* | 52 | EbpIII-PC | *Drosophila melanogaster (Dmel)* | FBpp0309999 | 42.743 | 6.57797e-05 | 43.2 | 64.9 |
| *OforOR1* | 307 | Or13a-PA | *Drosophila melanogaster (Dmel)* | FBpp0073938 | 44.669 | 0.000112595 | 23.9 | 39.7 |
| *OforOR2* | 346 | Orco-PB | *Drosophila melanogaster (Dmel)* | FBpp0112105 | 433.335 | 1.61449e-121 | 62 | 73.1 |
| *OforOR3* | 417 | Or2a-PA | *Drosophila melanogaster (Dmel)* | FBpp0070381 | 75.0998 | 1.50253e-13 | 24.5 | 45.6 |
| *OforOR4* | 111 | Orco-PB | *Drosophila melanogaster (Dmel)* | FBpp0112105 | 60.4622 | 3.01364e-10 | 34.1 | 60 |
| *OforOR5* | 479 | Or85d-PA | *Drosophila melanogaster (Dmel)* | FBpp0081361 | 80.4925 | 4.19937e-15 | 28.2 | 55 |
| *OforOR6* | 413 | Or49b-PA | *Drosophila melanogaster (Dmel)* | FBpp0086894 | 65.4698 | 1.13664e-10 | 22.8 | 41.1 |
| *OforOR7* | 286 | Or49b-PA | *Drosophila melanogaster (Dmel)* | FBpp0086894 | 73.1738 | 2.97715e-13 | 23.5 | 47.1 |
| *OforOR8* | 110 | Or43a-PA | *Drosophila melanogaster (Dmel)* | FBpp0088122 | 68.9366 | 8.70296e-13 | 38.7 | 69.3 |
| *OforOR9* | 275 | Or2a-PA | *Drosophila melanogaster (Dmel)* | FBpp0070381 | 80.4925 | 1.75329e-15 | 25.2 | 47.7 |
| *OforOR10* | 174 | Or49b-PA | *Drosophila melanogaster (Dmel)* | FBpp0086894 | 77.0258 | 8.98236e-15 | 24.4 | 53.6 |
| *OforOR11* | 121 | Or92a-PA | *Drosophila melanogaster (Dmel)* | FBpp0083295 | 60.077 | 4.48833e-10 | 25.2 | 52.3 |
| *OforOR12* | 124 | Or85f-PA | *Drosophila melanogaster (Dmel)* | FBpp0081506 | 57.7658 | 2.4431e-09 | 33.3 | 64.3 |

| | | | | | | | | |
|---|---|---|---|---|---|---|---|---|
| *OforOR13* | 96 | Or92a-PA | *Drosophila melanogaster (Dmel)* | FBpp0083295 | 43.8986 | 3.42232e-05 | 20.7 | 52.2 |
| *OforOR14* | 80 | KCNQ-PD | *Drosophila melanogaster (Dmel)* | FBpp0289111 | 29.6462 | 0.56352 | 25 | 50 |
| *OforOR15* | 157 | Or43a-PA | *Drosophila melanogaster (Dmel)* | FBpp0088122 | 65.855 | 1.54244e-11 | 26.2 | 48.1 |
| *OforGR1* | 273 | Gr64f-PA | *Drosophila melanogaster (Dmel)* | FBpp0073057 | 177.563 | 1.19045e-44 | 38 | 60.1 |
| *OforGR2* | 290 | Gr64a-PA | *Drosophila melanogaster (Dmel)* | FBpp0073050 | 164.851 | 7.50108e-41 | 35.6 | 56.2 |
| *OforGR3* | 308 | Gr64f-PA | *Drosophila melanogaster (Dmel)* | FBpp0073057 | 214.157 | 1.23227e-55 | 39.5 | 60.2 |
| *OforGR4* | 348 | Gr28b-PE | *Drosophila melanogaster (Dmel)* | FBpp0089143 | 46.595 | 4.39474e-05 | 26.1 | 51.3 |
| *OforGR5* | 248 | Gr63a-PB | *Drosophila melanogaster (Dmel)* | FBpp0113004 | 140.584 | 1.17536e-33 | 37.3 | 58.3 |
| *OforGR6* | 116 | Gr28a-PA | *Drosophila melanogaster (Dmel)* | FBpp0079087 | 78.5666 | 1.22593e-15 | 45.6 | 65.6 |
| *OforGR7* | 82 | Gr23a-PB | *Drosophila melanogaster (Dmel)* | FBpp0077386 | 41.2022 | 0.000194491 | 53.5 | 65.1 |
| *OforGR8* | 117 | Gr5a-PA | *Drosophila melanogaster (Dmel)* | FBpp0070768 | 43.5134 | 4.66392e-05 | 31.1 | 62.3 |
| *OforGR9* | 59 | Gr63a-PB | *Drosophila melanogaster (Dmel)* | FBpp0113004 | 35.8094 | 0.00822236 | 42.1 | 63.2 |
| *OforSNMP1* | 496 | Snmp1-PB | *Drosophila melanogaster (Dmel)* | FBpp0306056 | 359.762 | 2.98055e-99 | 39.3 | 60.5 |
| *OforSNMP2* | 515 | Snmp1-PB | *Drosophila melanogaster (Dmel)* | FBpp0306056 | 354.369 | 1.42787e-97 | 36.6 | 60.9 |
| *OforSNMP3* | 190 | Snmp2-PB | *Drosophila melanogaster (Dmel)* | FBpp0110185 | 92.8189 | 1.90631e-19 | 30.6 | 52.3 |
| *OforSNMP4* | 118 | Snmp2-PD | *Drosophila melanogaster (Dmel)* | FBpp0306711 | 86.6557 | 4.33878e-18 | 35.6 | 56.8 |

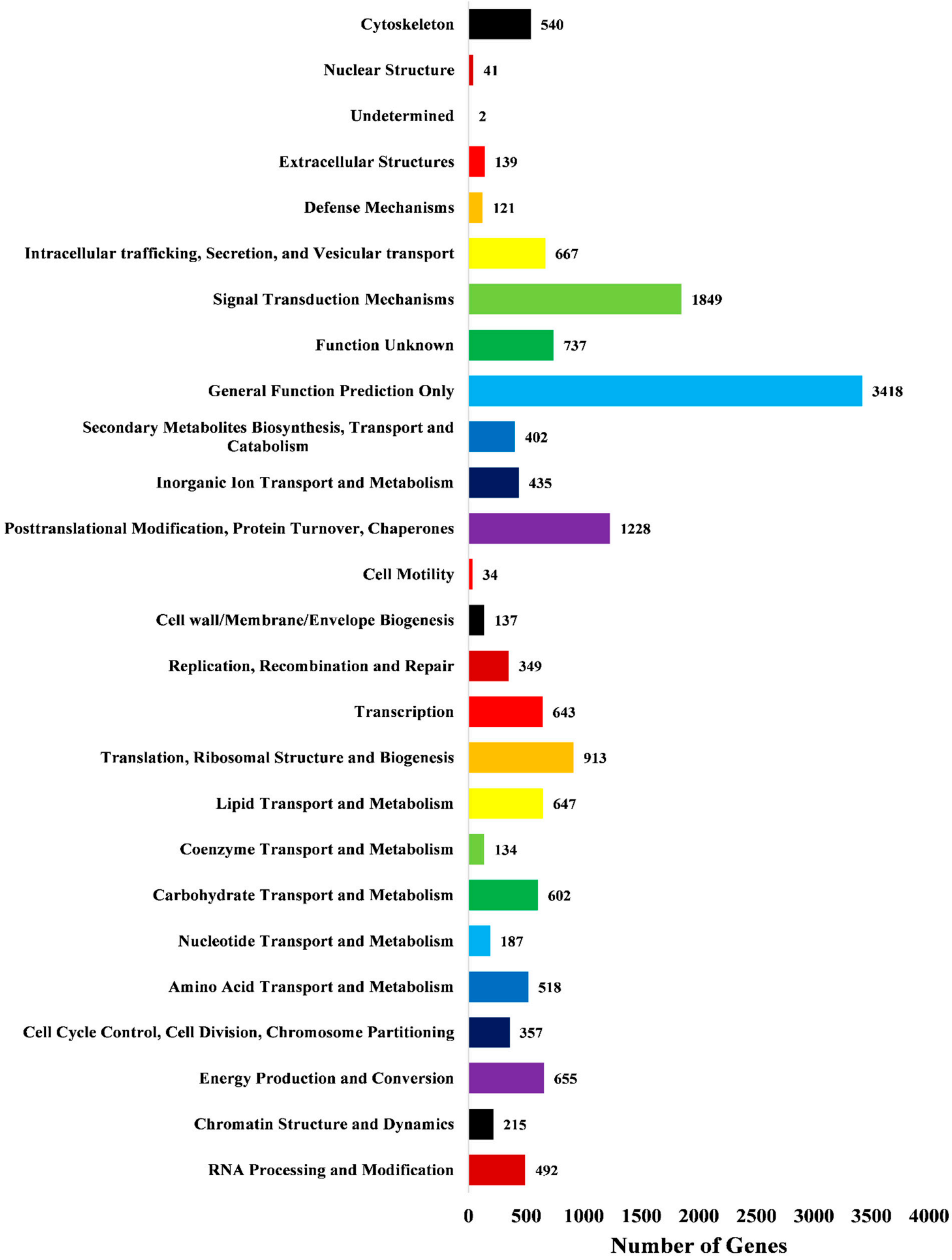


**Figure S1.** KOG annotation of unigenes in the *O. formosanus* workers.

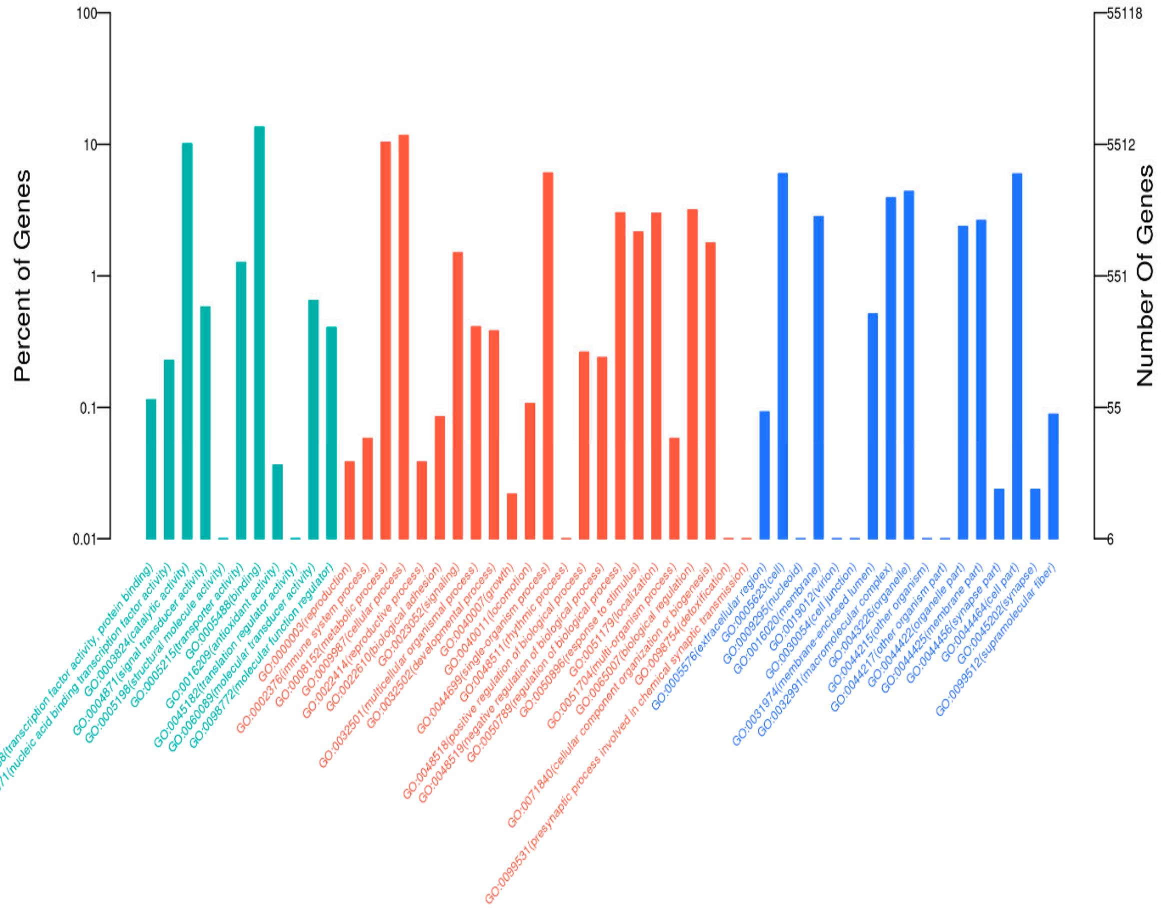


**Figure S2 .** Gene function categorization of *O. formosanus* worker unigenes using the Gene Ontology (GO) system.

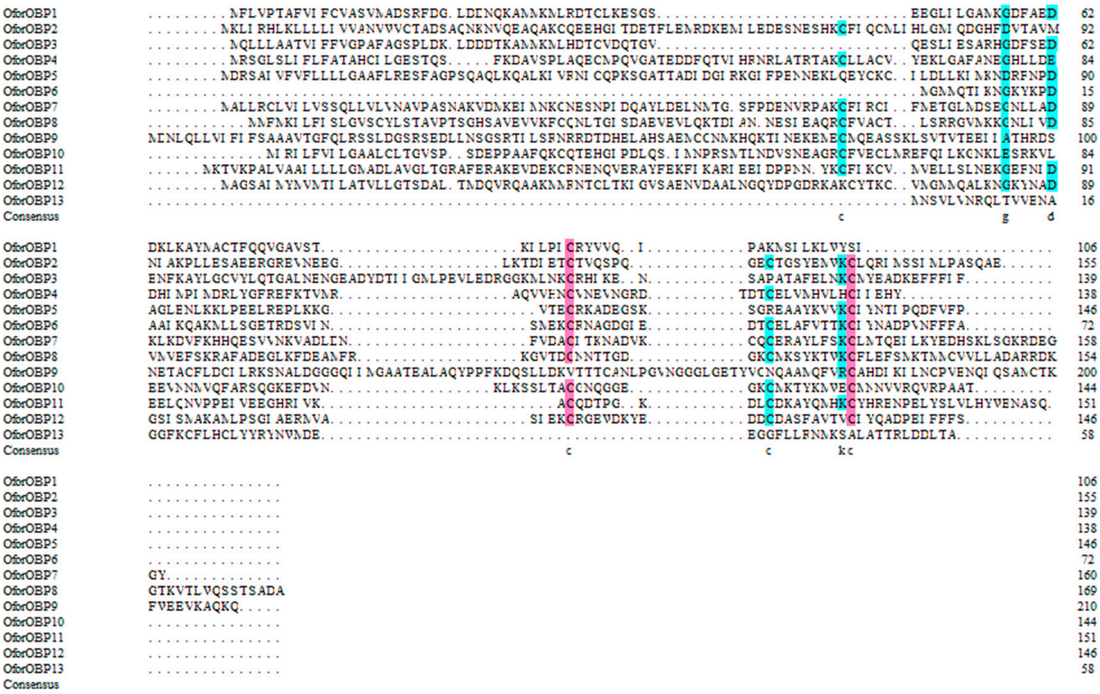

**Figure S3.** The multiple sequence alignment of *O. formosanus* putative OBPs. There is a 20.01% amino acid similarity between all the putative OBPs. Sea green and pink, highlights are applied to the same residues, signifying 80% and 90%, similarity, respectively.

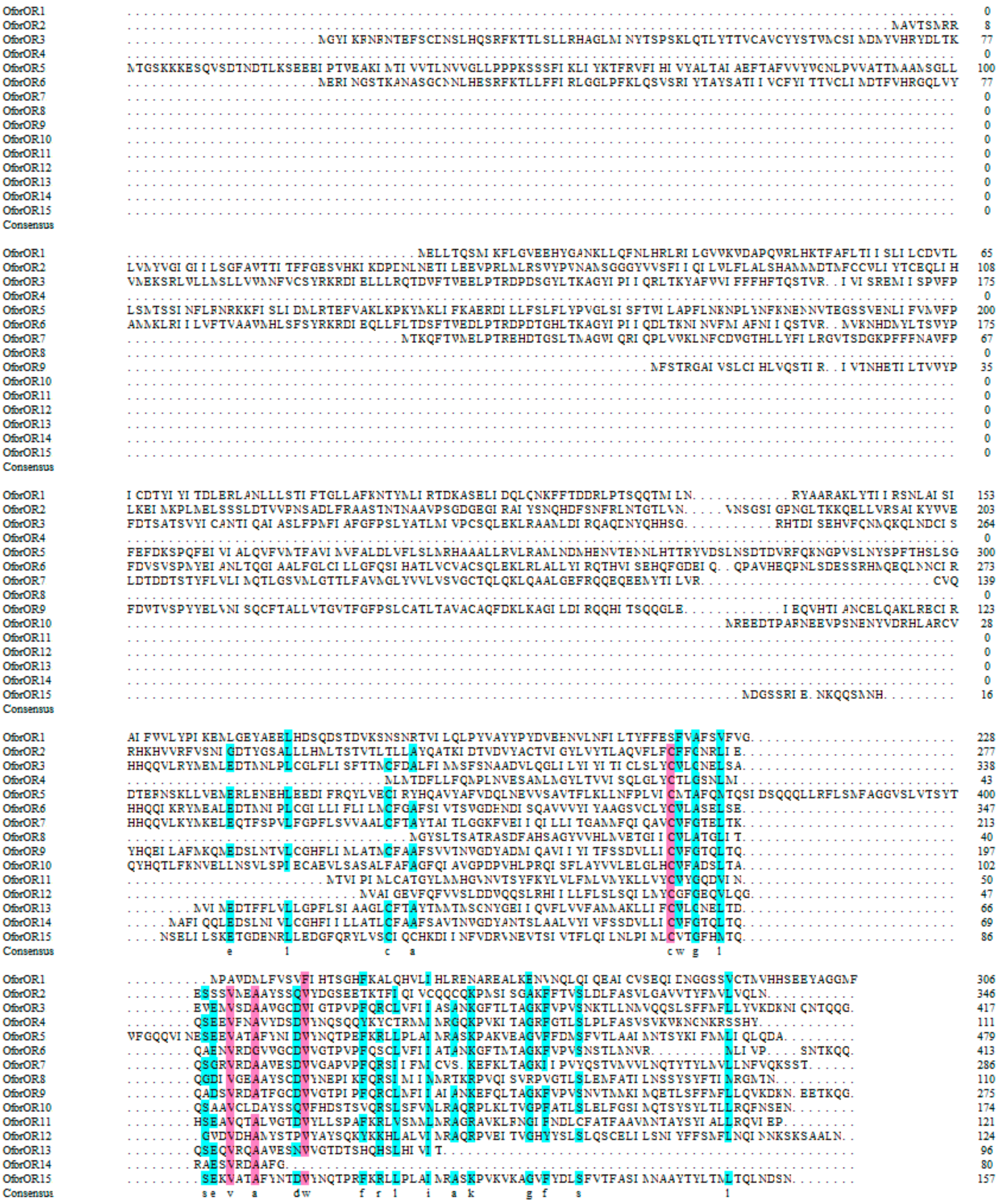

**Figure S4.** The multiple sequence alignment of *O. formosanus* putative ORs. There is a 18.65% amino acid similarity between all the putative ORs. Sea green and pink, highlights are applied to the same residues, signifying 80% and 90%, similarity, respectively.

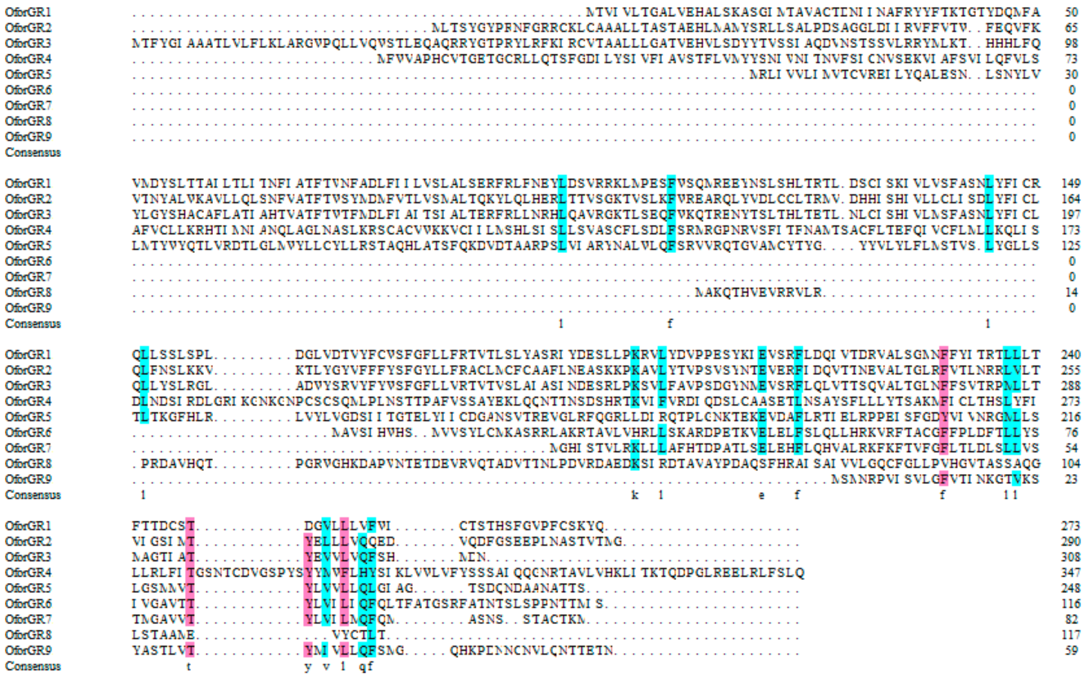

**Figure S5.** The multiple sequence alignment of *O. formosanus* putative GRs. There is a 21.36% amino acid similarity between all the putative GRs. Sea green and pink, highlights are applied to the same residues, signifying 80% and 90%, similarity, respectively.

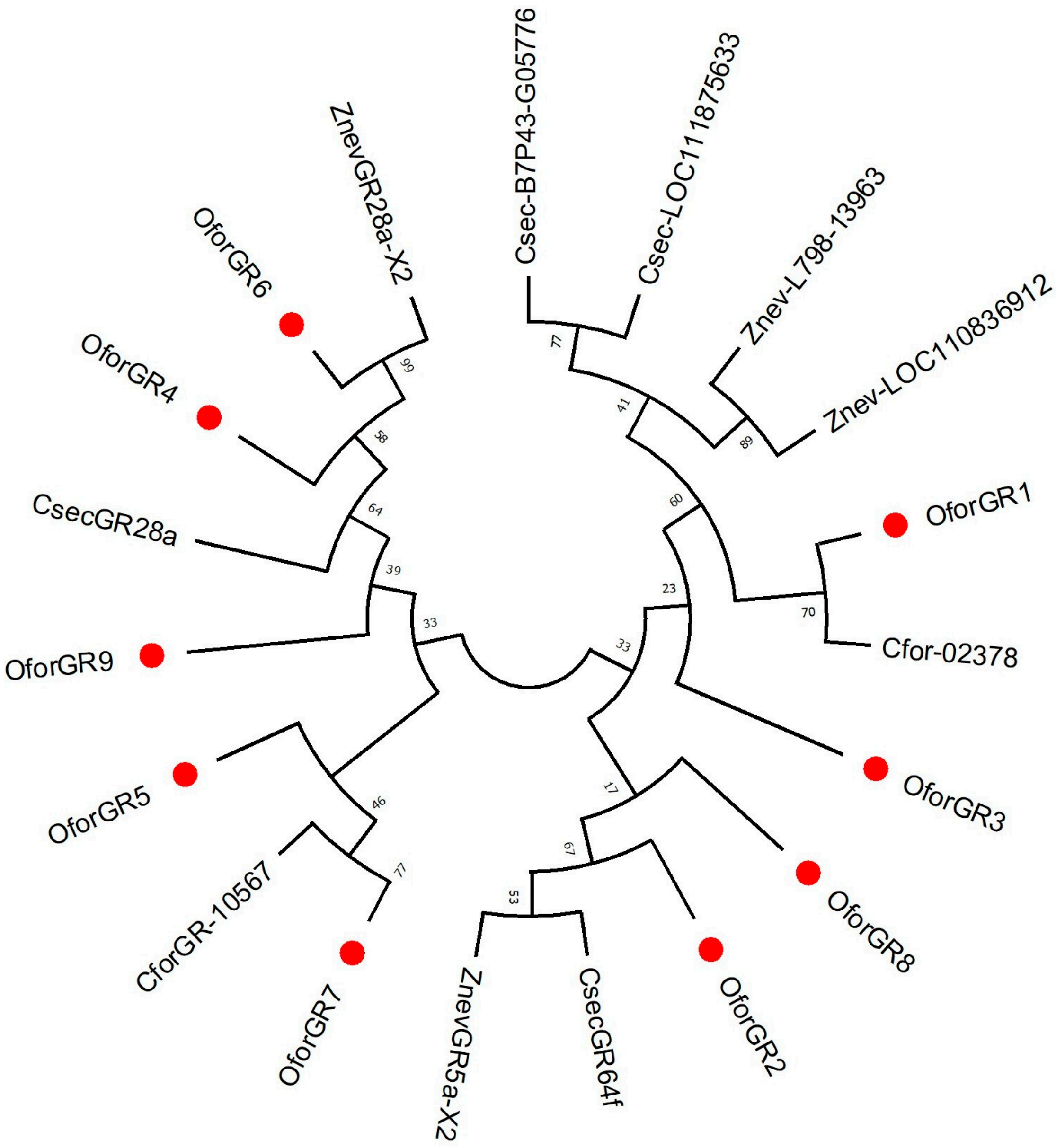


**Figure S6.** Phylogenetic tree of identified GRs with the GRs from different insect species. The identified GRs are marked red.

The percentages of bootstrap is shown in black color. The phylogenetic tree of 9 putative *OforGR*s was constructed by using the sequences from the insect species are; *Cryptotermes secundus, Zootermopsis nevadensis and Coptotermes formosanus*. GenBank accession number for all GRs genes are; Csec-B7P43-G05776; PNF40292.1, Znev-L798-13963; KDR11637.1, Cfor-02378; GFG38492.1, Csec-LOC111875633; XP_033606047.1, Znev-LOC110836912; XP_021934317.1, CsecGR64f; XP_023711366.1, ZnevGR5a-X2; XP_021936907.1, CsecGR28a; XP_023704213.2, ZnevGR28a-X2; XP_021920101.1, CforGR-10567; GFG40019.1.

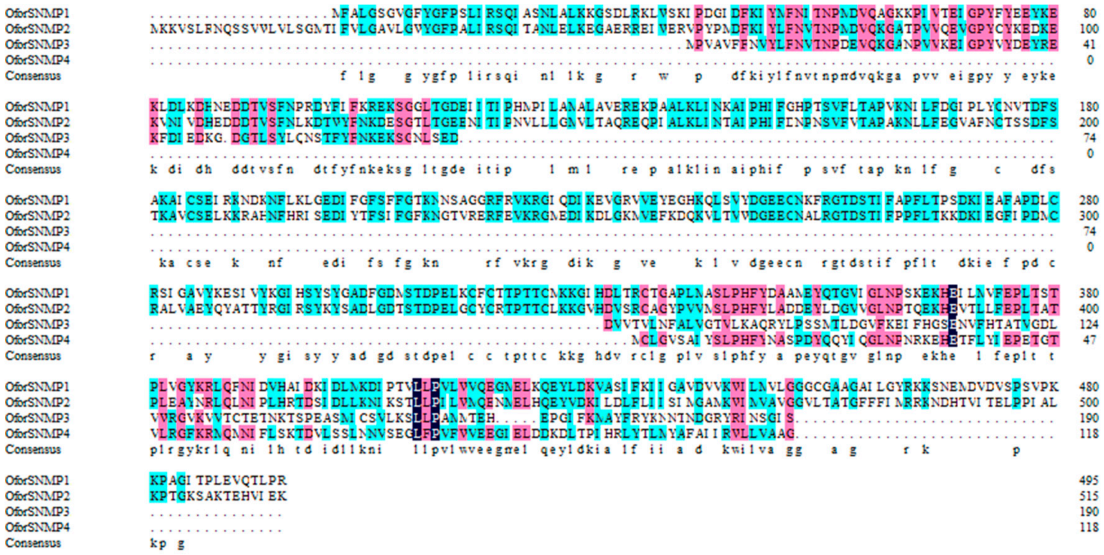

**Figure S7.** The multiple sequence alignment of *O. formosanus* putative SNMPs. There is a 33.62% amino acid similarity between all the putative SNMPs. Sea green, pink, and black highlights are applied to the same residues, signifying 80%, 90%, and 100% similarity, respectively.

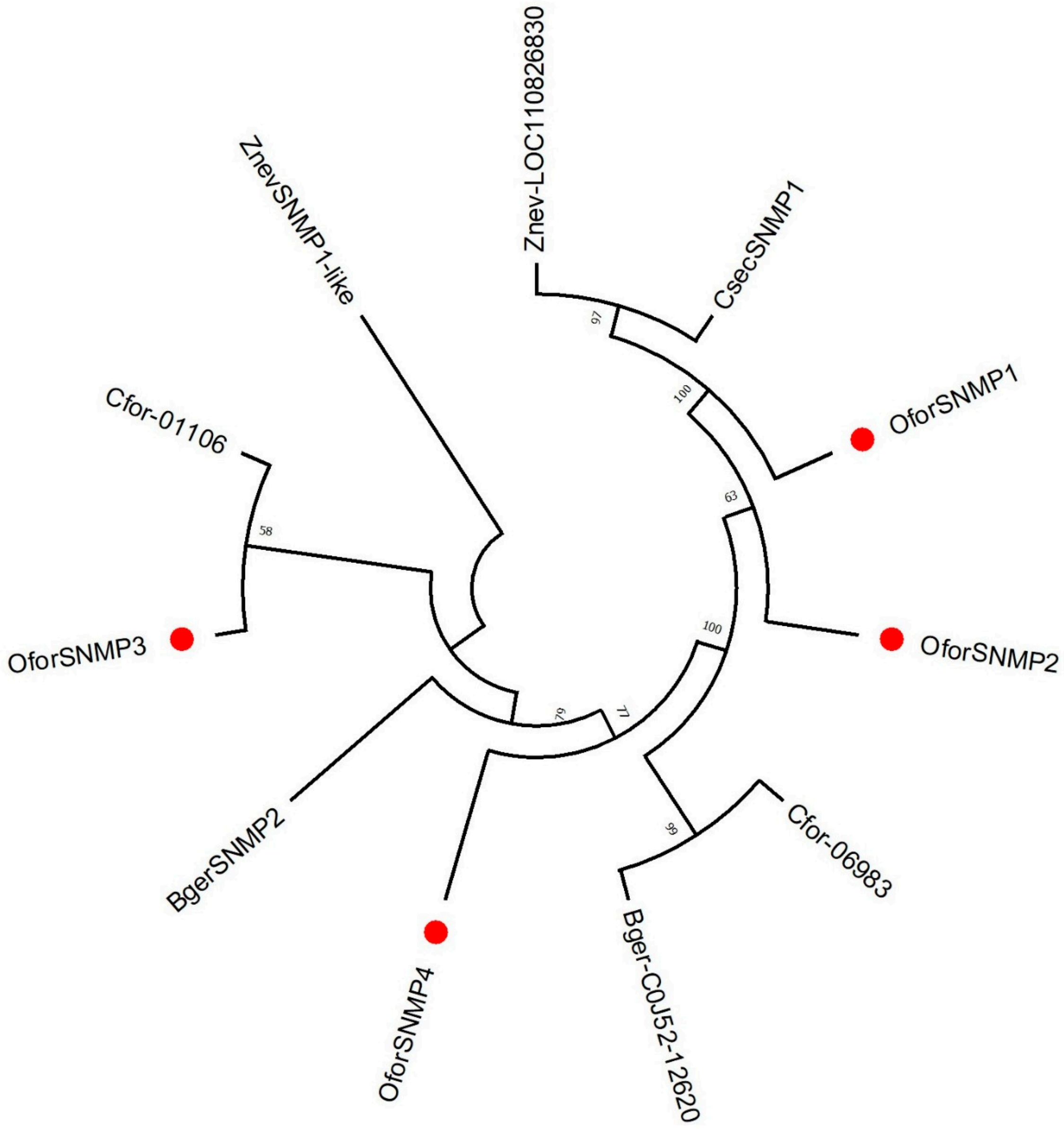


**Figure S8.** Phylogenetic tree of identified SNMPs with the SNMPs from different insect species. The identified SNMPs are marked red.

The percentages of bootstrap is shown in black color. The phylogenetic tree of 4 putative *OforSNMPs* was constructed by using the sequences from the insect species are; *Blattella germanica, Coptotermes formosanus, Zootermopsis nevadensis* and *Cryptotermes secundus*. GenBank accession number for all GRs genes are; BgerSNMP2; PSN50836.1, Cfor-01106; GFG35002.1, ZnevSNMP1-like; XP_021919116.1, Znev-LOC110826830; XP_021913553.1, Cfor-06983; GFG37744.1, Bger-C0J52-12620; PSN43241.1 and CsecSNMP1; XP_023702700.1.

**Gene Sequences;** Chemosensory genes identified in the transcriptome assembly of *O. formosanus*

***OforCSP***

***Odontotermes formosanus* Chemosensory Protein 1 (*OforCSP1*) (**OR651283)

**Nucleotide Sequence**

ATGTGCAGCAAATGTTCTCCTAGCCAGAAGAGTATGCTAAACGTAGCAGT

GCAGCACATTCAAACCAGCCCTCCAGAGCAGTGGAATCAGCTGCTGGCAA

CTTTCGATCCTGACGGGAAGAAAGCTGCAGAATTTATTGCTTATCTGGCG

AAACCTTAA

**Amino Acid Sequence**

MCSKCSPSQKSMLNVAVQHIQTSPPEQWNQLLATFDPDGKKAAEFIAYLA

KP *

***OforOBPs***

***Odontotermes formosanus* Odorant Binding Protein 1 (*OforOBP1*) (**OR651388)

**Nucleotide Sequence**

ATGTTTCTAGTTCCTACAGCTTTTGTCATTTTCTGTGTAGCCTCAGTCAT

GGCAGACTCTCGGTTTGATGGACTGGACGACAATCAGAAGGCGATGATGA

AAATGCTGCGCGACACTTGCCTGAAGGAAAGCGGCTCAGAGGAAGGTCTC

ATTCTAGGAGCCATGAAAGGCGACTTTGCCGAAGACGACAAGTTAAAGGC

GTACATGGCATGTACCTTCCAGCAAGTTGGAGCGGTAAGTACAAAAATCT

TACCCATCTGCAGGTACGTAGTTCAGATCCCAGCCAAGATGTCTATTCTG

AAATTGTGGTACAGTATCTGA

**Amino Acid Sequence**

MFLVPTAFVIFCVASVMADSRFDGLDDNQKAMMKMLRDTCLKESGSEEGL

ILGAMKGDFAEDDKLKAYMACTFQQVGAVSTKILPICRYVVQIPAKMSIL

KLWYSI *

***Odontotermes formosanus*** **Odorant Binding Protein 2 (*OforOBP2*) (**OR651389)

**Nucleotide Sequence**

ATGAAATTAATAAGACATCTGAAGTTACTTCTATTGATTGTTGTGGCCAA
CGTCTGGGTCTGCACTGCGGATTCGGCCCAAAACAAAAACGTACAGGAGG
CCCAAGCAAAGTGCCAGGAGGAACACGGAATAACTGATGAAACATTTTTG
GAAATGCGGGACAAAGAAATGATTCTGGAGGATGAATCAAACGAATCTCA
CAAGTGTTTCATACAATGCATGCTGATTCATCTTGGAATGATACAGGACG
GCCATTTTGACGTCACAGCGGTGATGAATATTGCCAAACCGCTGCTGGAA
AGTGCTGAAGAACGTGGTCGAGAGGTGAATGAAGAGGGTCTTAAGACGGA
CATTGAAACGTGTACCGTTCAAAGCCCACAAGGTGAATGCACAGGCAGTT
ATGAAATGTGGAAATGTCTGCAACGCATAATGTCCAGTATTATGTTGCCA
GCATCTCAGGCAGAATAA

**Amino Acid Sequence**

MKLIRHLKLLLLIVVANVWVCTADSAQNKNVQEAQAKCQEEHGITDETFL
EMRDKEMILEDESNESHKCFIQCMLIHLGMIQDGHFDVTAVMNIAKPLLE
SAEERGREVNEEGLKTDIETCTVQSPQGECTGSYEMWKCLQRIMSSIMLP
ASQAE *

***Odontotermes formosanus*** **Odorant Binding Protein 3 (*OforOBP3*) (**OR651390)

**Nucleotide Sequence**

ATGCAGTTGCTACTTGCCGCAACAGTCATCTTTTTTGTGGGCCCAGCTTT
TGCAGGTTCTCCGTTGGACAAACTTGATGACGATACAAAGGCTATGATGA
AAATGCTGCATGACACCTGTGTGGACCAAACTGGCGTGCAAGAGAGTCTA
ATCGAAAGTGCTAGACACGGGGACTTCAGCGAAGATGAAAATTTCAAGGC
CTATTTGGGATGCGTCTACCTACAAACAGGAGCGCTGAATGAAAATGGAG
AGGCAGACTATGACACCATCATCGGGATGTTGCCAGAAGTCTTAGAGGAC
CGCGGTGGCAAAATGCTGAACAAATGCAGACATATCAAAGAGAACAGTGC
TCCAGCTACGGCCTTTGAACTGAACAAGTGCATGTACGAAGCAGATAAAG
AGTTCTTCTTCATCTTCTAG

**Amino Acid Sequence**

MQLLLAATVIFFVGPAFAGSPLDKLDDDTKAMMKMLHDTCVDQTGVQESL

IESARHGDFSEDENFKAYLGCVYLQTGALNENGEADYDTIIGMLPEVLED

RGGKMLNKCRHIKENSAPATAFELNKCMYEADKEFFFIF *

***Odontotermes formosanus* Odorant Binding Protein 4 (*OforOBP4*)** (OR651391)

**Nucleotide Sequence**

ATGAGGAGCGGTTTAAGTCTCATCTTTCTGTTTGCCACTGCTCACTGTAT

TCTGGGAGAATCGACCCAGAGCTTTAAGGATGCTGTGTCACCACTAGCAC

AAGAGTGCATGCCGCAGGTTGGGGCGACAGAAGATGACTTCCAGACGGTG

ATCCACAGGAACCGCCTGGCCACTCGGACCGCCAAGTGTCTCCTGGCCTG

TGTCTACGAAAAGTTGGGCGCATTCGCAAATGAAGGTCACTTACTGGACG

AAGACCACATCATGCCTATTATGGACAGGCTGTATGGATTTAGAGAATTC

AAGACCGTGATGAGGGCCCAAGTGGTACACAACTGTGTCAATGAAGTGAA

CGGAAGAGACACTGACACATGTGAACTGGTGATGCACGTCCTGCACTGTA

TAATCGAGCACTACTGA

**Amino Acid Sequence**

MRSGLSLIFLFATAHCILGESTQSFKDAVSPLAQECMPQVGATEDDFQTV

IHRNRLATRTAKCLLACVYEKLGAFANEGHLLDEDHIMPIMDRLYGFREF

KTVMRAQVVHNCVNEVNGRDTDTCELVMHVLHCIIEHY *

***Odontotermes formosanus* Odorant Binding Protein 5 (*OforOBP5*) (**OR651392)

**Nucleotide Sequence**

ATGGACCGCAGTGCGATAGTTTTCGTCTTTCTGCTGCTTCTGGGTGCTGC

TTTCCTGCGGGAAAGCTTTGCGGGGCCGAGCCAAGCCCAATTGAAGCAAG

CGCTGAAAATTGTACGAAATATCTGTCAGCCGAAATCTGGAGCCACCACA

GCCGACATCGACGGAATTAGGAAAGGAATATTTCCCGAAAACAATGAAAA

ACTTCAGGAATACTGCAAGTGCATCCTTGACCTCTTAAAAATTATGAAGA

ACGACAGATTTAATCCGGATGCTGGTTTGGAGAATTTAAAAAAGCTGCCC

GAAGAATTGCGTGAACCCCTGAAAAAAGGTGTCACCGAGTGCCGCAAAGC

AGACGAAGGATCCAAATCGGGAAGAGAAGCTGCATACAAAGTCGTCAAGT

GCATTTACAATACAATACCACAAGACTTCGTATTCCCGTGA

**Amino Acid Sequence**

MDRSAIVFVFLLLLGAAFLRESFAGPSQAQLKQALKIVRNICQPKSGATT

ADIDGIRKGIFPENNEKLQEYCKCILDLLKIMKNDRFNPDAGLENLKKLP

EELREPLKKGVTECRKADEGSKSGREAAYKVVKCIYNTIPQDFVFP *

***Odontotermes formosanus* Odorant Binding Protein 6 (*OforOBP6*) (**OR651393)

**Nucleotide Sequence**

ATGGGAATGATGCAGACGATAAAGAACGGAAAGTACAAGCCGGACGCTGC

CATAAAACAAGCCAAGATGCTGCTGAGTGGTGAAACCAGGGACAGTGTGA

TTAATTCGATGGAGAAGTGCCGAAACGCGGGGGATGGAATAGAAGACACC

TGTGAATTGGCCTTCGTCACAACAAAGTGCATTTACAATGCAGATCCTGT

GAATTTCTTCTTCGCTTGA

**Amino Acid Sequence**

MGMMQTIKNGKYKPDAAIKQAKMLLSGETRDSVINSMEKCRNAGDGIEDT

CELAFVTTKCIYNADPVNFFFA*

***Odontotermes formosanus* Odorant Binding Protein 7 (*OforOBP7*) (**OR651394)

**Nucleotide Sequence**

ATGGCGCTGCTTCGCTGCTTGGTCATCCTAGTATCTTCTCAGCTCTTGGT
GCTGGTGAATGCAGTACCAGCGTCTAACGCTAAGGTGGACATGAAAGAAA
TTATGAATAAATGCAATGAGAGTAACCCTATAGACCAAGCCTACCTGGAT
GAGTTAAACATGACTGGCAGTTTTCCCGATGAGAACGTTAGACCTGCCAA
GTGTTTCATTCGGTGTATTTTTATGGAAACTGGACTGATGGATTCTGAAG
GCAACTTGCTAGCTGACAAGTTGAAGGATGTCTTCAAACATCACCAGGAA
TCTGTGGTCAACAAAGTCGCCGACCTGGATAATTTTGTGGATGCTTGTAT
AACTAAGAATGCCGATGTGAAATGCCAGTGCGAGAGGGCTTATCTCTTCT
CCAAGTGCCTGATGACACAGGAGATATTGAAATACGAAGACCACAGCAAG
TTGAGTGGTAAGAGAGATGAAGGGGGATACTGA

**Amino Acid Sequence**

MALLRCLVILVSSQLLVLVNAVPASNAKVDMKEIMNKCNESNPIDQAYLD
ELNMTGSFPDENVRPAKCFIRCIFMETGLMDSEGNLLADKLKDVFKHHQE
SVVNKVADLDNFVDACITKNADVKCQCERAYLFSKCLMTQEILKYEDHSK
LSGKRDEGGY *

***Odontotermes formosanus* Odorant Binding Protein 8 (*OforOBP8*) (**OR651395)

**Nucleotide Sequence**

ATGATGTTCATGAAAATTTTGTTTATTTCTCTTGGTGTCTCCTGCTACCT
TTCAACTGCTGTGCCTACAAGCGGCCATTCAGCTGTAGAAGTCGTGAAGT
TTTGCCAGAATCTAACAGGGATAAGTGACGCGGAAGTTGAGGTTCTTCAG
AAAACGGATATAGCAAATAATGAGTCCATAGAGGCGCAGAGGTGTTTTGT
GGCTTGCACGCTTAGCAGAAGAGGAGTGATGAAGAAGGGGAATCTTATCG
TTGACGTCATGGTTGAATTTTCGAAAAGAGCGTTTGCAGACGAGGGCCTA
AAGTTCGACGAAGCAATGTTCAGAAAGGGCGTTACCGATTGCAACAACAC
AACTGGAGACGGCAAATGTATGAAGAGCTACAAGACATGGAAATGCTTCC
TGGAATTTTCAATGAAGACAATGATGTGTGTGGTTTTATTAGCAGACGCA

AGAAGAGATAAAGGGACCAAAGTAACCTTATGGCAGTCTTCTACGTCTGC
AGATGCCGATTAA

**Amino Acid Sequence**

MMFMKILFISLGVSCYLSTAVPTSGHSAVEVVKFCQNLTGISDAEVEVLQ
KTDIANNESIEAQRCFVACTLSRRGVMKKGNLIVDVMVEFSKRAFADEGL
KFDEAMFRKGVTDCNNTTGDGKCMKSYKTWKCFLEFSMKTMMCVVLLADA
RRDKGTKVTLWQSSTSADAD *

***Odontotermes formosanus* Odorant Binding Protein 9 (*OforOBP9*)** (OR651396)

**Nucleotide Sequence**

ATGGACAACTTACAGCTACTGGTAATTTTCATTTTCAGCGCTGCTGCTGT
AACAGGCTTCCAGCTGAGATCCAGTCTCGATGGTTCCAGATCAGAAGATT
TATTAAACAGCGGTAGTAGGACAATACTGTCCAGAAACAGGAGAGACACT
GACCACGAACTGGCACACAGTGCGGAGATGTGCTGCAATATGAAGCATCA
AAAGACTATCAATGAAAAGGAAATGGAATGTATGCAGGAAGCCAGTTCTA
AATTGTCAGTAACAGTCACTGAAGAAATAATTGCAACCCACAGGGATTCC
AACGAGACAGCTTGTTTTTGGACTGTATTCTACGCAAAAGCAATGCACT
GGATGGCGGTGGACAGATAATAATGGGCGCAGCTACTGAAGCCTTAGCAC
AGTACCCTCCCTTTAAGGATCAAAGCTTACTCGATAAAGTGACAACGACT
TGTGCGAATTTACCTGGAGTAAACGGCGGCGGGCTTGGAGAGACCTACGT
GTGTAACCAAGCAGCAATGCAATTCGTGAGGTGTGCGCATGACATCAAGA
TACTTAACTGTCCGGTAGAGAATCAGATTCAATCTGCAATGTGTACGAAA
TTTTGGGAGGAAGTAAAGGCACAGAAGCAGTGA

**Amino Acid Sequence**

MDNLQLLVIFIFSAAAVTGFQLRSSLDGSRSEDLLNSGSRTILSRNRRDT
DHELAHSAEMCCNMKHQKTINEKEMECMQEASSKLSVTVTEEIIATHRDS
NETACFLDCILRKSNALDGGGQIIMGAATEALAQYPPFKDQSLLDKVTTT
CANLPGVNGGGLGETYVCNQAAMQFVRCAHDIKILNCPVENQIQSAMCTK
FWEEVKAQKQ *

***Odontotermes formosanus* Odorant Binding Protein 10 (*OforOBP10*)** (OR651397)

**Nucleotide Sequence**

ATGATACGGATATTATTTGTAATCCTCGGGGCCGCTCTTTGTCTAACTGG
AGTCTCTCCTTCGGATGAACCGCCCGCTGCTTTTCAAAAGTGCCAAACCG
AGCATGGAATTCCTGACCTTCAAAGTATCATGAATCCCCGATCAATGACG
CTGAATGACGTCTCAAATGAGGCTGGCAGGTGCTTCGTGGAGTGTCTAAT
GAGAGAATTTCAAATTCTTAAGGGGAACAAGCTTGAGAGCCGTAAGGTGT
TAGAAGAGGTTAACAATATGGTACAGTTCGCCAGATCGCAAGGAAAAGAG
TTTGATGTGAACAAACTCAAGTCCTCACTGACAGCATGCGGTAACCAAGG
TGGTGAGGGGAAGTGCATGAAGACTTACAAGATGTGGGAATGCATGAACA
ATGTTGTTCGACAGGTGAGACCAGCAGCTACATAA

**Amino Acid Sequence**

MIRILFVILGAALCLTGVSPSDEPPAAFQKCQTEHGIPDLQSIMNPRSMT
LNDVSNEAGRCFVECLMREFQILKGNKLESRKVLEEVNNMVQFARSQGKE
FDVNKLKSSLTACGNQGGEGKCMKTYKMWECMNNVVRQVRPAAT *

***Odontotermes formosanus* Odorant Binding Protein 11 (*OforOBP11*)** (OR651398)

**Nucleotide Sequence**

ATGAAGACTGTGAAACCTGCACTAGTGGCGGCCATCTTGCTGTTGCTGGG
AATGGCAGACCTCGCTGTGGGGTTGACAGGCCGTGCTTTTGAAAGAGCCA
AGGAAGTCGATGAGAAATGTAGAAACGAAAACCAAGTTGAGAGAGCGTAC
TTTGAAAAATTTATTAAAGCACGTATTGAAGAAATAGATCCTCCAAACAA
TTACAAGTGTTTTATTAAATGCGTCATGGTGGAACTATTATCTCTAAACG
AAAAGGGAGAATTCAACATCGACGAAGAACTGCAGAATGTACCACCGGAG
ATTGTGGAAGAGGGACATCGTATAGTCAAGGCTTGCCAAGACACACCCGG
AAAGGACTTATGTGACAAGGCTTACCAGATGCATAAGTGTTACCATAGAG
AAAATCCGGAGCTGTATTCGCTTGTGTTACATTACTGGGAAAATGCATCC
CAATAG

**Amino Acid Sequence**

MKTVKPALVAAILLLLGMADLAVGLTGRAFERAKEVDEKCRNENQVERAY
FEKFIKARIEEIDPPNNYKCFIKCVMVELLSLNEKGEFNIDEELQNVPPE
IVEEGHRIVKACQDTPGKDLCDKAYQMHKCYHRENPELYSLVLHYWENAS
Q *

***Odontotermes formosanus* Odorant Binding Protein 12 (*OforOBP12*) (**OR651399)

**Nucleotide Sequence**

ATGGCTGGGTCAGCAATCATGTATATGGTAATGACAATTTTGGCCACCGT
ACTGTTGGGGACATCTGATGCACTGACTATGGACCAAGTGCGGCAAGCGG
CAAAAATGATGAGAAATACATGCTTGACGAAAATAGGAGTCAGTGCAGAA
AATGTGGATGCTGCTTTAAATGGTCAGTATGATCCTGGTGACAGAAAAGC
TAAGTGTTACACGAAATGTGTCATGGGGATGATGCAGGCGTTGAAGAATG
GAAAGTACAATGCTGATGGTTCCATTTCAATGGCAAAGGCGATGCTGCCC
AGCGGTATAGCTGAAAGGATGGTCGCTTCCATCGAGAAGTGCCGCGGTGA
ATGGGACAAATATGAGGACGACTGCGATGCGTCTTTCGCAGTAACAGTGT
GCATATACCAAGCTGATCCCGAGATTTTCTTCTTCTCTTAA

**Amino Acid Sequence**

MAGSAIMYMVMTILATVLLGTSDALTMDQVRQAAKMMRNTCLTKIGVSAE
NVDAALNGQYDPGDRKAKCYTKCVMGMMQALKNGKYNADGSISMAKAMLP
SGIAERMVASIEKCRGEWDKYEDDCDASFAVTVCIYQADPEIFFFS *

***Odontotermes formosanus* Odorant Binding Protein 13 (*OforOBP13*) (partial) (**OR651400)

**Nucleotide Sequence**

ATGAACTCCGTACTGGTTAACAGGCAGCTGACCGTTGTGGAAAATGCAGG
GGGCTTCAAATGTTTCTTGCATTGCCTTTATTACAGGTACAACTGGATGG
ATGAAGAGGGAGGATTCCTTCTGCGCAACATGAAAAGCGCTTTGGCGACT
ACCAGACTGGATGATCTCACTGCT

**Amino Acid Sequence**

MNSVLVNRQLTVVENAGGFKCFLHCLYYRYNWMDEEGGFLLRNMKSALAT
TRLDDLTA

***OforORs***

***Odontotermes formosanus*** **Odorant receptor 1 (*OforOR1*)** (OR651429)

**Nucleotide Sequence**

ATGGAGCTACTAACACAAAGTATGATTAAATTCCTTGGTGTAGAGGAACA
TTATGGGGCAAACAAATTGCTTCAATTCAATCTTCACAGGCTACGTATTC
TGGGTGTCTGGAAGTGGGATGCGCCTCAGTGGAGACTGCATAAAACATTT
GCATTTTTAACTATTATATCACTGATTCTGTGTGATGTAACACTAATTTG
TGATACCTACATCTACATCACTGACTTAGAACGCCTAGCAAATTTACTCT
TAAGTACTATATTCACTGGCCTTCTTGCATTCAAGAATACTTACATGCTT
ATTAGAACTGATAAGGCTTCAGAACTTATTGATCAACTGCAAAACAAATT
TTTCACGGATGACCGACTGCCCACATCACAGCAGACAATGATCTTGAATA
GATACGCAGCAAGAGCAAAGCTCTATACTATCATAAGAAGCAATTTGGCA
ATCAGTATAGCTATATTCTGGGTTTTATATCCAATTAAGGAAATGTTGGG
GGAGTATGCAGAAGAACTACATGACTCACAGGACAGTACTGATGTGAAAT
CTAACAGCAACAGAACTGTCATTTTGCAGTTGCCATATGTTGCGTACTAT
CCTTACGATGTGGAACATAATGTTCTTAATTTCATTCTGACATACTTCTT
TGAGTCATTTGTTGCATTTTCTGTTTTTGTTGGAATGCCTGCTTGGGATA
TGCTTTTTGTTTCTGTATTTATTCATACTTCTGGGCATTTTAAAGCACTG
CAACATGTGTTGATCCATTTGCGTGAAAACGCTAGAGAAGCACTGAAAGA
AAATGTGAATCAACTTCAAATCCAGGAAGCCATTTGTGTGTCAGAACAAA
TTGACAATGGTGGCAGCAGTGTGTGCACAATGGTACATCATTCAGAAGAA
TATGCAGGAGGTATGTTTTTTTAG

**Amino Acid Sequence**

MELLTQSMIKFLGVEEHYGANKLLQFNLHRLRILGVWKWDAPQWRLHKTF
AFLTIISLILCDVTLICDTYIYITDLERLANLLLSTIFTGLLAFKNTYML
IRTDKASELIDQLQNKFFTDDRLPTSQQTMILNRYAARAKLYTIIRSNLA
ISIAIFWVLYPIKEMLGEYAEELHDSQDSTDVKSNSNRTVILQLPYVAYY
PYDVEHNVLNFILTYFFESFVAFSVFVGMPAWDMLFVSVFIHTSGHFKAL
QHVLIHLRENAREALKENVNQLQIQEAICVSEQIDNGGSSVCTMVHHSEE
YAGGMFF*

***Odontotermes formosanus* Odorant receptor 2 (*OforOR2*)** (OR651430)

**Nucleotide Sequence**

ATGGCAGTCACTAGCATGAGGCGTCTGGTCATGTACGTGGGCATCGGCAT
CATCCTCAGCGGGTTTGCCTGGACCACCATCACCTTCTTCGGAGAGAGTG
TGCACAAAATAAAGGACCCAGATAATTTGAATGAGACTATTTTAGAGGAG
GTACCGCGTCTCATGCTACGTTCGTGGTATCCCTGGAATGCTATGTCTGG
CGGAGGATACGTCGTGTCCTTCATCATCCAGATCCTGTGGCTATTTCTGG
CTCTGTCACATGCCATGATGATGGACACTATGTTCTGCTGTTGGCTCATC
TACACGTGTGAGCAACTCATTCATCTGAAAGAGATCATGAAGCCGCTGAT
GGAGCTCAGTTCATCGCTGGACACTGTGGTGCCTAACTCCGCAGACCTGT
TCCGTGCCGCCAGCACCAATACCAACGCTGCTGTACCTTCAGGTGATGGT
GAGGGTATTCGAGCTATATACAGCAACCAGCACGACTTCTCCAATTTCCG
ACTGAATACAGGCACCCTTGTCAACGTAAACAGTGGCTCTATTGGACCAA
ACGGACTGACAAAGAAACAGGAGCTCCTAGTACGGTCCGCCATCAAGTAC
TGGGTGGAGAGACACAAACACGTGGTTCGATTCGTCAGTAATATTGGAGA
CACGTACGGGTCGGCACTGCTGCTACATATGCTGACCAGCACAGTGACAT
TGACACTGCTCGCCTACCAGGCCACGAAGATTGACACTGTAGATGTCTAT
GCCTGCACCGTGATCGGCTATCTAGTGTACACATTGGCCCAGGTGTTTCT
CTTCTGCTTCTTTGGCAACCGTCTCATCGAGGAGAGCTCATCAGTGATGG
AGGCTGCCTACAGCAGTCAGTGGTATGACGGGTCGGAGGAAACCAAGACT

TTTATCCAGATTGTATGTCAACAATGTCAGAAACCAATGAGCATCTCTGG
AGCCAAATTCTTCACAGTGTCGCTCGATTTGTTTGCTTCGGTTCTTGGTG
CTGTAGTGACCTACTTCATGGTGCTGGTACAGCTCAACTAG

**Amino Acid Sequence**

MAVTSMRRLVMYVGIGIILSGFAWTTITFFGESVHKIKDPDNLNETILEE
VPRLMLRSWYPWNAMSGGGYVVSFIIQILWLFLALSHAMMMDTMFCCWLI
YTCEQLIHLKEIMKPLMELSSSLDTVVPNSADLFRAASTNTNAAVPSGDG
EGIRAIYSNQHDFSNFRLNTGTLVNVNSGSIGPNGLTKKQELLVRSAIKY
WVERHKHVVRFVSNIGDTYGSALLLHMLTSTVTLTLLAYQATKIDTVDVY
ACTVIGYLVYTLAQVFLFCFFGNRLIEESSSVMEAAYSSQWYDGSEETKT
FIQIVCQQCQKPMSISGAKFFTVSLDLFASVLGAVVTYFMVLVQLN*

***Odontotermes formosanus*** **Odorant receptor 3 (*OforOR3*) (**OR651431)

**Nucleotide Sequence**

ATGGGATACATCAAGAGGAACAGGAATACTGAGTTCTCGTGTGATAACAG
CCTTCATCAGAGCAGATTTAAGACGACTCTCTCTCTTTTGCGACACGCAG
GATTGATGATTAACTACACATCGCCATCAAAACTTCAAACTTTATACACT
ACAGTTTGTGCAGTGTGTTATTACAGTACTTGGATGTGTTCTATTATGGA
TATGTATGTTCACAGATATGATTTGACGAAAGTTATGGAGAAAAGTCGTT
TATGGCTTCTTATGTCACTCCTAGTGTGGATGAATTTCGTTTGCAGTTAC
CGTAAACGGGATATCGAACTGCTGCTCAGACAGACAGACTGGTTTACGTG
GGAAGAACTGCCTACCAGAGATCCTGACAGCGGATATTTGACAAAGGCTG
GGTACATCCCGATCATCCAACGTCTTACCAAATATGCTTTTTGGGTCATC
TTTTTTTTCCACTTCACCCAGTCAACAGTCCGCATTGTGATCAGCCGCGA
AATGATCATAAGTCCGTGGTTTCCGTTCGACACATCGGCGACTTCAGTAT
ACATATGTGCGAATACAATACAGGCCATTGCGTCACTCTTCCCAATGTTT
ATAGCTTTCGGCTTTCCCAGCCTGTACGCTACGCTAATGATTGTACCCTG
CAGTCAGCTGGAGAAGCTCAGAGCAGCCATGTTGGATATCAGGCAGGCAC
AAGACAATTATCAACATCATTCAGGGCGACATACAGACATTTCGGAACAC

GTGTTCCAAAATATGCAGAAACAACTAAACGACTGTATATCTCACCACCA
GCAAGTACTACGATACATGGAAATGTTGGAAGACACAATGAATCTTCCAC
TCTGTGGACTATTCCTGATTTCCTTCACAACGATGTGTTTCGACGCTCTC
TTCATTATGATGAGTTTCAGCAATGCTGCAGATGTACTGCAGGGTCTGAT
ATTGTATATTTATATAACTATCTGCTTGTCCCTCTACTGTTGGCTTGGAA
ACGAGCTATCAGCTGAGTGGGAGATGGTGAGTGACGCAGCCTGGGGCTGC
GACTGGATAGGAACTCCTGTCCCATTTCAGCGCTGTCTGGTCTTCATCAT
TGCCTCAGCTAATAAGGGGTTCACACTGACAGCTGGCAAATTTGTTCCGG
TCTCCAACAAAACATTGTTGAATATGGTGCAGCAATCTCTATCCTTCTTC
ATGTTTCTTCTATATGTGAAAGACAAGAATATCCAGAACACTCAGCAAGG
ATAA

**Amino Acid Sequence**

MGYIKRNRNTEFSCDNSLHQSRFKTTLSLLRHAGLMINYTSPSKLQTLYT
TVCAVCYYSTWMCSIMDMYVHRYDLTKVMEKSRLWLLMSLLVWMNFVCSY
RKRDIELLLRQTDWFTWEELPTRDPDSGYLTKAGYIPIIQRLTKYAFWVI
FFFHFTQSTVRIVISREMIISPWFPFDTSATSVYICANTIQAIASLFPMF
IAFGFPSLYATLMIVPCSQLEKLRAAMLDIRQAQDNYQHHSGRHTDISEH
VFQNMQKQLNDCISHHQQVLRYMEMLEDTMNLPLCGLFLISFTTMCFDAL
FIMMSFSNAADVLQGLILYIYITICLSLYCWLGNELSAEWEMVSDAAWGC
DWIGTPVPFQRCLVFIIASANKGFTLTAGKFVPVSNKTLLNMVQQSLSFF
MFLLYVKDKNIQNTQQG*

***Odontotermes formosanus* Odorant receptor 4 (*OforOR4*)** (OR651432)

**Nucleotide Sequence**

ATGCTTATGACAGATTTCTTACTGTTCCAGATGCCGCTCAATGTGGAATC

TGCGATGCTAATGGGATATTTAACAGTTGTGATTTCTCAGTTGGGCCTGT

ATTGTACATTAGGGTCCAATTTAATGATCCAGAGCGAGGAGGTGTTCAAC

GCCGTGTACGACAGTGACTGGTACAACCAGTCGCAACAATACAAATACTG

CACCAGGATGATGATCATGAGAGGCCAGAAGCCGGTGAAAATCACCGCTG

GCAGGTTCGGAACTTTGTCCCTTCCCCTCTTTGCTTCGGTGAGTGTAAAG

TGGAAAAATGGTAATAAACGTAGCTCGCACTATTAG

**Amino Acid Sequence**

MLMTDFLLFQMPLNVESAMLMGYLTVVISQLGLYCTLGSNLMIQSEEVFN

AVYDSDWYNQSQQYKYCTRMMIMRGQKPVKITAGRFGTLSLPLFASVSVK

WKNGNKRSSHY*

***Odontotermes formosanus* Odorant receptor 5 (*OforOR5*)** (OR651433)

**Nucleotide Sequence**

ATGACTGGATCAAAGAAGAAAGAAAGTCAAGTTTCTGATACAAATGACAC

ATTAAAAAGTGAAGAAGAAATTCCAACGTGGGAAGCGAAAATCATGACTA

TCGTCGTCACCCTGAATGTAGTTGGTCTCCTTCCTCCTCCCAAATCTTCG

TCATTCATCAAATTGATCTACAAAACTTTCAGAGTATTCATACATATAGT

TTACGCGTTGACAGCGATAGCGGAGTTCACTGCCTTCGTGGTTTACTGGG

GAAATCTTCCCGTAGTTGCCACCACTATGGCTGCCATGTCTGGTTTACTC

TTATCAATGACCTCTTCCATAAATTTTCTGCGCAACAGAAAGAAATTCAT

AAGCCTTATAGATATGCTGAGAACAGAATTTGTTGCCAAATTGAAACCAA

AGTATATGAAACTTATTTTTAAGGCTGAACGTGACATTTTACTCTTTTCA

TTATTCTTATACCCTGTAGGTTTAAGTATTTCTTTTACTTGGATATTAGC

GCCCTTTTTAAATAAGAACCCCCTATATAACTTTAAAAATGAAAATAATG

TCACAGAAGGAAGTAGTGTGGAGAATTTAATTTTTGTGATGTGGTTCCCT

TTTGAATTTGACAAGTCCCCACAATTTGAAATAGTAATAGCGTTGCAGGT

TTTTGTTATGACTTTTGCGGTAATAATGGTATTTGCACTTGATTTAGTGT
TTCTGTCTCTGATGAGACATGCCGCTGCTCTGTTAAGGGTGTTGCGTGCC
ATGCTGAATGATATGCATGAGAACGTCACAGAAAACAATTTGCATACAAC
AAGGTACGTAGATTCTTTGAACAGTGACACTGATGTCAGATTCCAGAAGA
ACGGCCCAGTGTCACTAAATTATTCCCCCTTTACCCATTCTTTGAGCGGA
GATACAGAGAGAAATTCAAAGCTTCTTGTTGAGATGGAACGCCTGGAAAA
TGAACACTTGGAGGAAGATATTTTCCGGCAGTACCTAGTTGAATGCATCA
GATATCACCAGGCTGTATACGCGTTTGTTGATCAACTGAACGAGGTTGTA
AGTGCTGTAACTTTCTTGAAGTTACTTAACTTTCCGTTAGTGATATGTAT
GACAGCATTTCAGATGACGCAGTCGATAGACAGCCAGCAGCAGTTACTGA
GATTCCTCTCCATGTTCGCTGGGGGTGTCTCTCTAGTAACGTCATATACG
TGGTTCGGACAGCAAGTAATTAACGAGAGCGAGGAAGTAGCGACAGCTTT
TTACAACATTGACTGGTACAACCAGACGCCAGAGTTCAAACGTCTGCTAC
CTCTAGCCATCATGCGCGCGTCGAAACCCGCCAAAGTTGAGGCCGGAGTG
TTCTTTGACATGTCTTTTGTCACTTTAGCTGCGATTATGAATACGTCTTA
TAAGATTTTCATGATGCTAATCCAACTGCAAGACGCCTAA

**Amino Acid Sequence**

MTGSKKKESQVSDTNDTLKSEEEIPTWEAKIMTIVVTLNVVGLLPPPKSS
SFIKLIYKTFRVFIHIVYALTAIAEFTAFVVYWGNLPVVATTMAAMSGLL
LSMTSSINFLRNRKKFISLIDMLRTEFVAKLKPKYMKLIFKAERDILLFS
LFLYPVGLSISFTWILAPFLNKNPLYNFKNENNVTEGSSVENLIFVMWFP
FEFDKSPQFEIVIALQVFVMTFAVIMVFALDLVFLSLMRHAAALLRVLRA
MLNDMHENVTENNLHTTRYVDSLNSDTDVRFQKNGPVSLNYSPFTHSLSG
DTERNSKLLVEMERLENEHLEEDIFRQYLVECIRYHQAVYAFVDQLNEVV
SAVTFLKLLNFPLVICMTAFQMTQSIDSQQQLLRFLSMFAGGVSLVTSYT
WFGQQVINESEEVATAFYNIDWYNQTPEFKRLLPLAIMRASKPAKVEAGV
FFDMSFVTLAAIMNTSYKIFMMLIQLQDA*

***Odontotermes formosanus* Odorant receptor 6 (*OforOR6*) (partial) (**OR651434)

**Nucleotide Sequence**

ATGGAGCGTATCAACGGTAGCACTAAGGCTAACGCGTCAGGCTGTAATAA
TCTACACGAAAGTCGTTTTAAGACACTTCTCTTTTTCATTCGACTGGGCG
GACTTCCATTTAAGTTACAGTCAGTGTCAAGAATTTACACAGCATACAGT
GCAACCATTATCGTGTGTTTTTACATTACTACTGTATGTCTTATCATGGA
TACGTTCGTTCATAGAGGCCAACTGGTGTACGCTATGATGAAATTGCGCA
TAATTCTTGTATTCACAGTAGCTGCATGGATGCACCTCAGTTTCAGTTAT
CGCAAACGTGATATCGAGCAACTTCTTTTTCTGACCGATTCCTTCACGTG
GGAGGACCTGCCTACCAGAGATCCTGACACCGGGCATTTAACAAAGGCTG
GGTACATACCGATCATCCAGGATCTTACTAAAAACATAAATGTATTCATG
ATTGCATTCAACATTATCCAGAGTACTGTGCGCATGGTGAAGAACCATGA
TATGTACCTCACGAGTTGGTACCCTTTTGACGTGTCAGTGAGTCCAATGT
ATGAAATCGCAAATCTCACACAGGGCATAGCAGCACTATTCGGATTATGT
ATATTACTTGGGTTCCAAAGCATACACGCTACGCTAGTCTGTGTAGCCTG
CAGCCAGCTGGAGAAGCTCAGACTGGCGCTGTTGTATATCAGACAGACAC
ACGTCATATCAGAACATCAATTCGGAGATGAGATACAACAGCCAGCTGTT
CACGAACAACCAAACCTTTCTGATGAATCGTCCCGTCACATGCAGGAACA
ACTTAACAACTGCATACGTCACCACCAGCAGATAAAGCGATACATGGAAG
CACTAGAAGACACAATGAATATCCCTCTATGCGGTATACTGCTCATTTTC
CTAATACTAATGTGCTTTGGCGCTTTCTCCATCGTCACGAGTTGGGGAGA
TCACAACGATATATCGCAGGCTGTAGTAGTGTACATTTATGCAGCGGGAT
CTGTATGTTTATACTGCTGGCTTGCGAGTGAGCTGTCAGAGCAGGCAGAA
AATGTAAGAGACGGTGTCTGGGGCTGCGACTGGGTAGGAACTCCTGTCCC
ATTTCAGAGCTGTCTGGTCTTCATCATTGCTACAGCCAACAAGGGGTTCA
CAATGACAGCTGGGAAATTTGTTCCAGTGTCCAACTCTACATTGATGAAT
GTAAGAATGCTAATCGTTCCGTCTAATACGAAGCAGCAG

**Amino Acid Sequence**

MERINGSTKANASGCNNLHESRFKTLLFFIRLGGLPFKLQSVSRIYTAYS
ATIIVCFYITTVCLIMDTFVHRGQLVYAMMKLRIILVFTVAAWMHLSFSY
RKRDIEQLLFLTDSFTWEDLPTRDPDTGHLTKAGYIPIIQDLTKNINVFM
IAFNIIQSTVRMVKNHDMYLTSWYPFDVSVSPMYEIANLTQGIAALFGLC
ILLGFQSIHATLVCVACSQLEKLRLALLYIRQTHVISEHQFGDEIQQPAV
HEQPNLSDESSRHMQEQLNNCIRHHQQIKRYMEALEDTMNIPLCGILLIF
LILMCFGAFSIVTSWGDHNDISQAVVVYIYAAGSVCLYCWLASELSEQAE
NVRDGVWGCDWVGTPVPFQSCLVFIIATANKGFTMTAGKFVPVSNSTLMN
VRMLIVPSNTKQQ

***Odontotermes formosanus*** **Odorant receptor 7 (*OforOR7*)** (OR651435)

**Nucleotide Sequence**

ATGACCAAACAGTTCACGTGGATGGAACTTCCAACCAGGGAGCACGACAC
TGGCTCACTCACCATGGCCGGCTGGATTCAGCGTATCCAGCCTTTGGTGT
GGAAATTAAATTTCTGCGACTGGGGGACTCACTTACTATACTTCATCCTG
CGTGGTGTTACAAGTGATGGCAAACCGTTTTTCTTTAACGCCTGGTTCCC
GCTTGATACCGACGACACTTCTACTTACTTTCTTGTACTCATCATGCAGA
CTCTGGGCAGTGTGATGCTAGGCACAACACTGTTCGCGGTGATGGGACTG
TATGTGGTGCTGGTATCTGTGGGCTGCACCCAGCTGCAGAAATTGCAGGC
GGCATTGGGCGAATTCAGGCAGCAGGAACAAGAAGAGATGTACACCATTC
TGGTCAGATGTGTGCAACACCATCAGCAAGTTCTGAAATACATGAAGGAG
CTAGAGCAAACATTCAGCCCTGTACTCTTCGGTCCTTTCCTGTCGGTGGT
GGCAGCACTCTGTTTTACCGCCTACACTGCAATAACACTGGGCGGAAAGT
TTGTGGAGATTATACAGATCCTCCTCATTACCGGTGCCATGATGTTTCAG
ATACAGGCTGTGTGTTGGTTTGGTACTGAGCTAACTAAACAGTCTGGCAG
AGTGCGGGATGCAGCATGGGAGAGCGACTGGGTGGGTGCGCCTGTTCCCT
TCCAGCGGTCCATCATATTCATGATTTGCGTCTCAAAGGAATTCAAACTC

ACAGCTGGCAAGATTATCCCCGTGTATCAAAGCACTGTCATGGTGGTGCT
GAACCAGACCTATACATATCTGATGGTACTCCTGAACTTTGTGCAAAAAT
CCAGTACATAA

**Amino Acid Sequence**

MTKQFTWMELPTREHDTGSLTMAGWIQRIQPLVWKLNFCDWGTHLLYFIL
RGVTSDGKPFFFNAWFPLDTDDTSTYFLVLIMQTLGSVMLGTTLFAVMGL
YVVLVSVGCTQLQKLQAALGEFRQQEQEEMYTILVRCVQHHQQVLKYMKE
LEQTFSPVLFGPFLSVVAALCFTAYTAITLGGKFVEIIQILLITGAMMFQ
IQAVCWFGTELTKQSGRVRDAAWESDWVGAPVPFQRSIIFMICVSKEFKL
TAGKIIPVYQSTVMVVLNQTYTYLMVLLNFVQKSST*

***Odontotermes formosanus*** **Odorant receptor 8 (*OforOR8*) (**OR651436)

**Nucleotide Sequence**

ATGGGTTACAGTTTGACAAGTGCTACACGGGCATCAGATTTTGCACATTC
TGCAGGATATGTCGTACATCTTATGGTGGAGACCGGAATCATTTGCTGGC
TGGCCACTGGTCTTATTACACAGGGCGACATAGTTGGGGAAGCCGCGTAT
TCGTGTGACTGGTACAACGAACCAATCAAATTTCAACGCTCCATAATGAT
CATCATGATGCGCACGAAACGGCCCGTGCAGATATCAGTGCGACCTGTAG
GAACGCTATCCTTGGAGATGTTTGCTACCATCCTGAACTCCTCTTACTCA
TATTTCACTATTATGAGAGGCATGACAAACTGA

**Amino Acid Sequence**

MGYSLTSATRASDFAHSAGYVVHLMVETGIICWLATGLITQGDIVGEAAY
SCDWYNEPIKFQRSIMIIMMRTKRPVQISVRPVGTLSLEMFATILNSSYS
YFTIMRGMTN*

***Odontotermes formosanus* Odorant receptor 9 (*OforOR9*)** (OR651437)

**Nucleotide Sequence**

ATGTTCAGCACCCGTGGAGCTATTGTGTCACTATGTATTCATTTAGTCCA
ATCAACAATCCGCATCGTGACAAACCACGAAACGATACTAACTGTGTGGT
ATCCTTTCGACTGGACTGTTTCGCCGTACTACGAACTGGTTAACATCTCA
CAGTGCTTTACAGCATTACTAGTAACGGGTGTGACATTCGGTTTCCCGTC
TCTGTGCGCTACTCTGACTGCTGTGGCCTGTGCTCAGTTCGATAAGCTAA
AGGCGGGCATATTGGATATCAGACAGCAACATATCACATCTCAACAAGGG
CTGGAAATTGAACAAGTTCATACAATTGCAAACTGTGAATTGCAAGCCAA
GCTAAGGGAGTGTATTCGATACCATCAGGAAATATTGGCGTTCATGAAGC
AAATGGAAGACTCGCTAAACACCGTTCTGTGTGGCCACTTTCTGATTATG
CTGGCCACAATGTGCTTCGCTGCTTTTTCTGTAGTTACGAACTGGGGAGA
TTATGCAGATATGATTCAAGCAGTTATAATTTACATAACGTTCTCCAGTG
ACGTGCTTCTGATCTGCTGGTTTGGAACTCAGTTGACACAACAGGCAGAC
AGTGTGAGAGACGCTACATTCGGGTGCGACTGGGTGGGAACTCCTATTCC
ATTTCAGAGATGTCTAATGTTCATCATCGCTATAGCTAACAAGGAGTTCC
AACTGACAGCGGGAAAGTTTGTTCCTGTATCGAATGTGACCATGATGAAA
ATAATGCAAGAGACTCTGTCCTTCTTCATGTTTCTGCTTCAAGTGAAAGA
CAAGAATGAAGAAACGAAACAGGGTTAA

**Amino Acid Sequence**

MFSTRGAIVSLCIHLVQSTIRIVTNHETILTVWYPFDWTVSPYYELVNIS
QCFTALLVTGVTFGFPSLCATLTAVACAQFDKLKAGILDIRQQHITSQQG
LEIEQVHTIANCELQAKLRECIRYHQEILAFMKQMEDSLNTVLCGHFLIM
LATMCFAAFSVVTNWGDYADMIQAVIIYITFSSDVLLICWFGTQLTQQAD
SVRDATFGCDWVGTPIPFQRCLMFIIAIANKEFQLTAGKFVPVSNVTMMK
IMQETLSFFMFLLQVKDKNEETKQG*

***Odontotermes formosanus* Odorant receptor 10 (*OforOR10*)** (OR651438)

**Nucleotide Sequence**

ATGCGTGAAGAAGACACCCCGGCCAGAAACGAGGAGGTACCGAGCAATGA
AAATTATGTTGACCGCCACTTGGCGCGCTGTGTGCAGTACCATCAGACAC
TGTTCAAGAACGTGGAGCTGCTCAACTCCGTTCTGAGCCCCATTGAGTGC
GCAGAGGTTTTAAGTGCTTCGGCTCTCTTCGCGTTTGCTGGATTCCAGAT
TGCGGTTGGGCCCGACCCCGTGCATCTCCCCCGTCAGATCAGTTTCCTCG
CGTATGTCGTGCTGGAGCTGGGACTGCACTGCTGGTTTGCCGACAGCCTC
ACTGCTCAGAGCGCCGCTGTGTGCTTGGATGCGTATTCCAGCCAGTGGTT
CCACGATTCGACAAGCGTCCAGCGGTCCCTGTCTTTTGTCATGTTACGAG
CACAGCGCCCCCTCAAGCTGACTGTGGGCCCTTTCGCCACTCTCTCACTA
GAACTGTTCGGCTCGATTATGCAGACTTCCTATTCGTATCTGACACTGCT
GAGACAATTTAACTCCGAAAATTAG

**Amino Acid Sequence**

MREEDTPARNEEVPSNENYVDRHLARCVQYHQTLFKNVELLNSVLSPIEC
AEVLSASALFAFAGFQIAVGPDPVHLPRQISFLAYVVLELGLHCWFADSL
TAQSAAVCLDAYSSQWFHDSTSVQRSLSFVMLRAQRPLKLTVGPFATLSL
ELFGSIMQTSYSYLTLLRQFNSEN*

***Odontotermes formosanus* Odorant receptor 11 (*OforOR11*)** (OR651439)

**Nucleotide Sequence**

ATGACCGTCATCCCGATCATGCTGTGTGCCACAGGATACCTCATGATGCA
CGGTGTCAATGTCACCAGCTACTTCAAGTACCTCGTCCTGTTCATGCTGG
TCATGTACAAACTGCTGGTGTACTGCTGGTATGGCCAGGATGTTATCAAT
CACAGCGAAGCAGTGCAGACAGCGCTCTGGGGCACCGACTGGTACCTGCT
GTCGCCCGCCTTCAAGCGTCTGGTTTCCATGATGCTCATGAGGGCCGGCA
GGGCTGTCAAACTAAGGAACGGCATTTTCAACGATCTGTGTTTTGCGACC
TTCGCAGCGGTGATGAACACTGCATATTCGTACATCGCACTGCTACGACA
GGTCATCGAACCGTAA

**Amino Acid Sequence**

MTVIPIMLCATGYLMMHGVNVTSYFKYLVLFMLVMYKLLVYCWYGQDVIN

HSEAVQTALWGTDWYLLSPAFKRLVSMMLMRAGRAVKLRNGIFNDLCFAT

FAAVMNTAYSYIALLRQVIEP*

***Odontotermes formosanus*** **Odorant receptor 12 (*OforOR12*)** (OR651440)

**Nucleotide Sequence**

ATGGTGGCAATTGGAGAGGTCTTCCAGTTTGTGGTTAGTCTGGATGACTG

GCAGCAGAGCCTAAGGCACATCATTCTGCTCTTTCTGTCTCTGTCACAAA

TACTGATGTACTGCGGCTTCGGTGAGCAAGTGCTGCAGGGGGGGTGGGAT

GTGGACCACGCTATGTATTCAACACCATGGTACGCGTACTCGCAGAAGTA

CAAGAAACATCTGGCTCTGGTGATAATGCGCGCGCAGCGACCAGTGGAGA

TTACCGTTGGTCATTACTACAGCCTGTCACTGCAATCGTGTGAACTGATC

CTTTCGAACATATACTTCTTCTCCATGTTCCTGAACCAGATTAACAACAA

ATCAAAATCTGCTGCTCTCAATTGA

**Amino Acid Sequence**

MVAIGEVFQFVVSLDDWQQSLRHIILLFLSLSQILMYCGFGEQVLQGGWD

VDHAMYSTPWYAYSQKYKKHLALVIMRAQRPVEITVGHYYSLSLQSCELI

LSNIYFFSMFLNQINNKSKSAALN*

***Odontotermes formosanus*** **Odorant receptor 13 (*OforOR13*) (partial) (**OR651441)

**Nucleotide Sequence**

ATGGTCATAATGGAGGATACATTTTTTCTCGTATTGCTCGGACCCTTCTT

ATCTATAGCCGCCGGATTGTGTTTTACTGCGTACACAATGATGACGATGT

CTGGGAACTACGGTGAGATAATACAGGTGTTTCTAGTTGTTTTCGCAATG

ATGGCCAAGTTACTGATTTTCTGCTGGCTTGGAAATGAACTGACAGATCA

GTCTGAACAGGTCCGCCAAGCGGCGTGGGAAAGCAACTGGGTGGGGACAG

ATACCTCGCATCAACATTCTTTACACATTGTGATAACT

**Amino Acid Sequence**

MVIMEDTFFLVLLGPFLSIAAGLCFTAYTMMTMSGNYGEIIQVFLVVFAM

MAKLLIFCWLGNELTDQSEQVRQAAWESNWVGTDTSHQHSLHIVIT

***Odontotermes formosanus* Odorant receptor 14 (*OforOR14*) (partial)** (OR651442)

**Nucleotide Sequence**

ATGGCGTTCATTCAGCAACTGGAAGATTCGCTGAATATCGTCCTGTGTGG

TCACTTTATAATTTTACTGGCCACGTTGTGCTTCGCTGCTTTTTCTGCAG

TTACGAACTGGGGAGATTACGCAAATACGAGTCTAGCAGCTTTAGTTTAT

ATTGTGTTCTCTAGTGACGTGCTTCTGATATGCTGGTTTGGAACTCAGCT

TACACAACGGGCTGAGAGTGTGAGAGACGCTGCATTCGGG

**Amino Acid Sequence**

MAFIQQLEDSLNIVLCGHFIILLATLCFAAFSAVTNWGDYANTSLAALVY

IVFSSDVLLICWFGTQLTQRAESVRDAAFG

***Odontotermes formosanus* Odorant receptor 15 (*OforOR15*)** (OR651443)

**Nucleotide Sequence**

ATGGATGGAAGTTCGCGGATTGAGAACAAACAGCAGTCGATGAATCACAA

TTCAGAGCTGATTTTGTCAAAGGAAACTGGTGACGAAAATCGTCTGCTCG

AAGACGGATTCCAGAGATACCTGGTTAGCTGCATTCAATGTCACAAAGAC

ATAATTAATTTCGTAGATCGCGTAAATGAGGTGACAAGCATAGTGACTTT

CTTGCAAATACTCAACCTACCAATAATGTTGTGCGTCACAGGATTTCACA

TGACTCAGAGCGAAAAGGTAGCAACAGCTTTTTACAACACTGACTGGTAC

AACCAGACGCCAAGGTTCAAACGTCTACTGCCTCTAGCCATCATGCGCGC

CTCGAAACCTGTCAAAGTTAAAGCCGGCGTGTTCTACGATTTGTCTTTCG

TCACTTTCGCTTCGATTATGAACGCAGCTTACACCTATTTAACTATGCTG

ACACAACTCAATGATTCGAATTAA

**Amino Acid Sequence**

MDGSSRIENKQQSMNHNSELILSKETGDENRLLEDGFQRYLVSCIQCHKD

IINFVDRVNEVTSIVTFLQILNLPIMLCVTGFHMTQSEKVATAFYNTDWY

NQTPRFKRLLPLAIMRASKPVKVKAGVFYDLSFVTFASIMNAAYTYLTML

TQLNDSN*

***OforGRs***

***Odontotermes formosanus* Gustatory receptor 1 (*OforGR1*)** (OR651376)

**Nucleotide Sequence**

ATGACAGTGATCGTGTTGACTGGAGCCCTGGTGGAACATGCACTTAGTAA

GGCAAGTGGCATTATGACAGCAGTAGCATGCACAGACAACATAATCAATG

CTTTCCGCTACTACTTCACAAAGACTGGCACATATGACCAAATGTTTGCT

GTTATGGACTACAGTCTTACAACGGCCATTTTAACATTAATTACCAACTT

CATTGCCACATTCACATGGAACTTTGCAGATTTATTCATCATACTGGTGA

GCCTTGCGCTCAGTGAGAGATTTCGCCTCTTCAATGAGTACCTTGACTCA

GTCCGGCGGAAGTTGATGCCAGAAAGCTTCTGGAGCCAAATGAGAGAAGA

ATACAACAGTCTCTCACATCTCACCAGGACTCTTGATTCTTGCATTTCCA

AGATTGTGCTTGTGTCCTTTGCAAGTAACCTGTACTTCATTTGTCGTCAG

CTCCTGAGTAGTCTGAGCCCATTAGATGGTTTAGTAGACACTGTATACTT

CTGCTGGTCATTTGGCTTTCTGTTGTTCAGAACAGTGACATTGTCTCTGT

ATGCATCAAGAATATATGATGAAAGTCTGTTGCCAAAGAGGGTACTGTAT

GATGTTCCTCCAGAAAGCTACAAAATAGAGGTATCAAGATTTCTGGATCA

GATAGTGACAGACAGAGTGGCACTATCAGGAATGAACTTTTTCTACATCA

CAAGAACCCTGTTGTTGACATTTACCACAGATTGCAGTACAGATGGTGTA

CTTTTGCTTGTCTTTTGGATATGTACTTCTACGCATAGTTTCGGTGTCCC

TTTCTGCAGCAAGTATCAATGA

**Amino Acid Sequence**

MTVIVLTGALVEHALSKASGIMTAVACTDNIINAFRYYFTKTGTYDQMFA
VMDYSLTTAILTLITNFIATFTWNFADLFIILVSLALSERFRLFNEYLDS
VRRKLMPESFWSQMREEYNSLSHLTRTLDSCISKIVLVSFASNLYFICRQ
LLSSLSPLDGLVDTVYFCWSFGFLLFRTVTLSLYASRIYDESLLPKRVLY
DVPPESYKIEVSRFLDQIVTDRVALSGMNFFYITRTLLLTFTTDCSTDGV
LLLVFWICTSTHSFGVPFCSKYQ*

***Odontotermes formosanus* Gustatory receptor 2 (*OforGR2*) (**OR651377)

**Nucleotide Sequence**

ATGCTGACATCTTACGGTTATCCAAGGAATTTTGGGCGACGATGCAAGCT
GTGTGCTGCCGCACTGCTGACCGCCTCCACAGCCGAACACCTCATGGCTA
TGTATTCGCGTCTGCTGTCCGCTCTGCCAGATTCGGCGGGAGGTTTAGAT
ATTATCCGTGTCTTCTTCGTGACCTGGTTCGAGCAGGTATTTAAAGTGAC
AAACTACGCTCTCTGGAAGGCGGTGCTGCTGCAGCTGTCCAACTTCGTGG
CTACCTTTACGTGGAGCTACATGGATATGTTTGTGACCCTCGTGAGCATG
GCGCTCACACAGAAGTACCTGCAGCTGCACGAGCGACTGACGACCGTGAG
CGGCAAGACCGTTTCGTTGAAGTTCTGGCGCGAGGCTCGTCAGCTGTACG
TCGACCTGTGCTGCCTCACACGGATGGTTGACCATCACATCTCACACATA
GTGCTGCTGTGCCTGATCAGCGACCTCTACTTCATCTGCCTTCAGCTGTT
CAACAGCCTCAAGAAAGTGAAGACTTTATATGGATACGTTTTCTTCTTCT
ACTCTTTCGGCTACTTGCTGTTCCGAGCATGCTTGATGTGCTTCTGTGCT
GCATTCTTGAACGAGGCCAGCAAGAAACCCAAGGCAGTCCTGTACACAGT
GCCGTCCGTCAGCTATAACACGGAGGTTGAGCGATTTATCGATCAAGTGA
CGACCAATGAGGTGGCACTAACTGGACTTCGCTTCGTGACGTTAAACAGA
CGCTTAGTTTTGACTGTCATTGGTTCAATCATGACGTATGAACTGCTTCT
CGTTCAACAAGAAGACGTTCAGGATTTTGGCAGTGAAGAACCACTAAACG
CGTCCACCGTTACAATGGGTTGA

**Amino Acid Sequence**

MLTSYGYPRNFGRRCKLCAAALLTASTAEHLMAMYSRLLSALPDSAGGLD

IIRVFFVTWFEQVFKVTNYALWKAVLLQLSNFVATFTWSYMDMFVTLVSM

ALTQKYLQLHERLTTVSGKTVSLKFWREARQLYVDLCCLTRMVDHHISHI

VLLCLISDLYFICLQLFNSLKKVKTLYGYVFFFYSFGYLLFRACLMCFCA

AFLNEASKKPKAVLYTVPSVSYNTEVERFIDQVTTNEVALTGLRFVTLNR

RLVLTVIGSIMTYELLLVQQEDVQDFGSEEPLNASTVTMG*

***Odontotermes formosanus* Gustatory receptor 3 (*OforGR3*)** (OR651378)

**Nucleotide Sequence**

ATGACCTTCTACGGCATAGCAGCTGCCACTCTCGTCTTGTTCCTGAAGCT

GGCAAGGGGCTGGCCGCAGCTGCTGGTGCAGTGGAGCACCCTGGAACAGG

CTCAGCGCCGCTACGGCACACCACGGTACTTACGCTTCAAGATTCGGTGC

GTCACCGCCGCACTTCTCTTGGGAGCAACAGTTGAACACGTGCTCAGCGA

TTACTACACCGTGTCCTCTATAGCGCAGGATGTCAACTCCACCAGCAGTG

TCCTTCGCCGGTACATGCTGAAGACTCACCACCATTTGTTCCAGTACCTC

GGATACTCCCACGCATGCGCATTCTTGGCTACGATTGCCCATACTGTGGC

CACATTCACCTGGACCTTCATGGACCTGTTCATCGCCATTACCAGTATCG

CTCTGACAGAGCGCTTCCGTCTCTTGAACCGACATCTGCAGGCCGTCAGG

GGGAAGACGCTGTCCGAGCAGTTCTGGAAACAGACACGAGAGAACTACAC

CAGCCTGACGCATCTCACCGAGACGCTCAATCTCTGCATATCCCACATCG

TGCTCATGTCCTTTGCAAGCAATCTGTACTTCATCTGCCTCCAGCTCCTC

TATAGTTTGAGGGGGCTTGCCGACTGGTACTCGCGGGTCTACTTCTACTG

GTCGTTCGGGTTCCTCTTGGTACGAACAGTCACCGTTTCTCTCGCCATCG

CTAGCATCAACGACGAGAGTCGTCTGCCCAAGTCCGTCCTGTTTGCTGTT

CCTTCGGACGGCTACAACATGGAAGTGTCGCGGTTCCTACAGCTGGTCAC

CACGTCGCAAGTGGCGCTCACCGGCCTGAACTTCTTTTCCGTGACCCGCC

CAATGCTGCTGACGATGGCGGGTACCATCGCCACTTACGAGGTCGTGTTG

GTGCAGTTCAGTCACATGGACAACTAA

**Amino Acid Sequence**

MTFYGIAAATLVLFLKLARGWPQLLVQWSTLEQAQRRYGTPRYLRFKIRC
VTAALLLGATVEHVLSDYYTVSSIAQDVNSTSSVLRRYMLKTHHHLFQYL
GYSHACAFLATIAHTVATFTWTFMDLFIAITSIALTERFRLLNRHLQAVR
GKTLSEQFWKQTRENYTSLTHLTETLNLCISHIVLMSFASNLYFICLQLL
YSLRGLADWYSRVYFYWSFGFLLVRTVTVSLAIASINDESRLPKSVLFAV
PSDGYNMEVSRFLQLVTTSQVALTGLNFFSVTRPMLLTMAGTIATYEVVL
VQFSHMDN*

***Odontotermes formosanus* Gustatory receptor 4 (*OforGR4*) (partial) (**OR651379)

**Nucleotide Sequence**

ATGTTTTGGGTAGCACCACACTGCGTAACTGGAGAAACAGGCTGTCGCCT
GCTGCAAACTTCTTTTGGCGACATTTTATACAGCATCGTCTTCATCGCCG
TTTCTACTTTTCTTGTTATGTACTATTCCAACATCTGGAATATCACGAAC
GTGTTTTCCATTTGTAATGTCAGTGAGAAGGTAATAGCCTTCAGCGTCAT
TCTGCAGTTCGTGTTGTCGGCGTTTGTGTGTCTCCTAAAGCGGCACACAA
TAATGAATATTGCAAATCAGCTGGCAGGTCTTAACGCCTCACTCAAACGT
TCCTGTGCCTGTGTTTGGAAGAAGGTCTGCATTATCCTCATGTCTCATCT
CTCTATCAGCCTCCTGAGTGTAGCCAGTTGCTTTTTATCGGACCTGTTTT
CTCGCATGCGAGGTCCAAATCGTGTGTCATTTATAACCTTCAACGCAATG
ACTTCTGCCTGCTTTCTTACTGAGTTTCAAATCGTTTGTTTCTTGATGCT
TTTGAAGCAACTTATTTCCGACTTGAACGACTCCATTCGTGATCTTGGGA
GAATAAAAGGTAACAAAGGTAACCCCTGTTCTTGTTCCCAAATGTTACCA
CTGAACAGCACGACACCAGCGTTCGTCTCTTCAGCCTACGAAAAGCTGCA
ACAGAATACCACGAACAGCGATTCCCACAGAACAAAAGTGATTTTCGTAC
GAGATATTCAGGATTCTCTTTGTGCAGCCTCTGAGACACTGAACTCTGCA
TATTCTTTCCTGCTGCTCTACACCTCAGCGAAGATGTTCATCTGTCTTAC
CCACAGTCTCTACTTCATTCTCCTACGCCTCTTCATAACCGGCTCCAACA
CCTGTGACGTGGGGTCACCATATTCATATTACATGTGGTTCCTGCACTAC

TCCATCAAGCTGGTGTGGCTGGTCTTCTACAGCAGCTCGGCTATCCAGCA
GGGAAACCGCACTGCAGTTCTGGTGCACAAACTGATCACCAAGACGCAGG
ATCCTGGGCTGAGAGAGGAGCTTCGTCTGTTCTCACTTCAGCTC

**Amino Acid Sequence**

MFWVAPHCVTGETGCRLLQTSFGDILYSIVFIAVSTFLVMYYSNIWNITN
VFSICNVSEKVIAFSVILQFVLSAFVCLLKRHTIMNIANQLAGLNASLKR
SCACVWKKVCIILMSHLSISLLSVASCFLSDLFSRMRGPNRVSFITFNAM
TSACFLTEFQIVCFLMLLKQLISDLNDSIRDLGRIKGNKGNPCSCSQMLP
LNSTTPAFVSSAYEKLQQNTTNSDSHRTKVIFVRDIQDSLCAASETLNSA
YSFLLLYTSAKMFICLTHSLYFILLRLFITGSNTCDVGSPYSYYMWFLHY
SIKLVWLVFYSSSAIQQGNRTAVLVHKLITKTQDPGLREELRLFSLQL

***Odontotermes formosanus* Gustatory receptor 5 (*OforGR5*) (**OR651380)

**Nucleotide Sequence**

ATGAGGCTCATCGTCGTGTTGATCATGGTGACATGCGTACGGGAAATCTT
GTACCAGGCACTGGAAAGCAACCTGAGCAACTATTTGGTACTGATGACGT
ACTGGTACCAAACATTGGTACGTGACACGTTGGGTCTCATGTGGTACCTA
CTGTGCTACCTGCTACGAAGCACTGCTCAGCACCTGGCCACCAGCTTTCA
GAAGGATGTAGACACTGCTGCGCGCCCCAGCCTGGTAATCGCACGTTACA
ACGCGCTGTGGCTACAGTTCAGCCGCGTGGTACGTCAGACCGGGGTGGCT
ATGTGCTACACGTATGGCTACTACGTACTCTACCTGTTCCTCATGAGCAC
CGTGTCCCTGTACGGGCTGTTATCCACCCTGACAAAGGGCTTCCATTTGC
GCCTCGTCTACCTGGTGGGCGACAGCATTATTACTGGCACCGAGCTCTAC
ATCATCTGTGACGGTGCCAATTCCGTCACCCGAGAGGTGGGTCTAAGATT
CCAGGGGAGGCTGTTGGACATCAGACAGACACCATTGGGCAATAAAACGG
AGAAGGAGGTTGACGCCTTCTTGAGAACCATCGAACTGCGTCCGCCGGAA
ATCAGTTTCGGAGATTACGTCATCGTGAACAGAGGAATGCTACTGTCATT
GGGTTCCATGATGGTCACGTACCTCGTGGTCCTGCTGCAGTTGGGGATTG
CGGGAACATCCGATCAAAACGACGCTGCGAACGCTACTACGTCATAG

**Amino Acid Sequence**

MRLIVVLIMVTCVREILYQALESNLSNYLVLMTYWYQTLVRDTLGLMWYL

LCYLLRSTAQHLATSFQKDVDTAARPSLVIARYNALWLQFSRVVRQTGVA

MCYTYGYYVLYLFLMSTVSLYGLLSTLTKGFHLRLVYLVGDSIITGTELY

IICDGANSVTREVGLRFQGRLLDIRQTPLGNKTEKEVDAFLRTIELRPPE

ISFGDYVIVNRGMLLSLGSMMVTYLVVLLQLGIAGTSDQNDAANATTS*

***Odontotermes formosanus* Gustatory receptor 6 (*OforGR6*) (partial) (**OR651381)

**Nucleotide Sequence**

ATGGCGGTATCGATTCACTGGCACTCGATGGTGGTATCGTACCTCTGCAT

GAAAGCATCTAGGCGACTGGCGAAGCGCACTGCAGTGCTGGTACACAGGC

TACTGAGCAAGGCCAGGGATCCTGAGACCAAAGTGGAGCTTGAACTCTTC

TCGCTGCAGCTCCTACATCGGAAGGTCCGATTCACAGCCTGCGGGTTCTT

TCCTTTGGACTTCACACTGCTATACTCGATTGTCGGCGCTGTTACGACGT

ATCTGGTCATACTGATTCAGTTTCAACTCACGTTTGCGACTGGAAGCAGA

TTTGCGACAAATACGTCATTATCACCACCTAACACCACCATGATTTCC

**Amino Acid Sequence**

MAVSIHWHSMVVSYLCMKASRRLAKRTAVLVHRLLSKARDPETKVELELF

SLQLLHRKVRFTACGFFPLDFTLLYSIVGAVTTYLVILIQFQLTFATGSR

FATNTSLSPPNTTMIS

***Odontotermes formosanus* Gustatory receptor 7 (*OforGR7*)** (OR651382)

**Nucleotide Sequence**

ATGGGGCACATTTCAACTGTGCTTCGCAAACTACTGCTTGCATTTCATAC

TGATCCAGCAACTCTGTCTGAACTGGAACACTTCTTACAACACGTCGCTC

TACGTAAGTTCAAATTCACGGTATTCGGATTCCTCACCCTCGATTTGTCT

CTGCTAGTCTCCACGATGGGCGCCGTGGTCACGTATCTTGTGATCCTCAT

GCAGTTCCAAATGGCAAGTAACAGTTCAACTGCATGTACCAAAATGTGA

**Amino Acid Sequence**

MGHISTVLRKLLLAFHTDPATLSELEHFLQHVALRKFKFTVFGFLTLDLS
LLVSTMGAVVTYLVILMQFQMASNSSTACTKM*

***Odontotermes formosanus* Gustatory receptor 8 (*OforGR8*) (partial) (**OR651383)

**Nucleotide Sequence**

ATGGCGAAGCAAACGCACGTTGAAGTCCGCAGAGTCCTGAGACCACGTGA
CGCCGTGCACCAGACGCCTGGCCGCTGGGGACACAAGGACGCTCCTTGGA
ACACGGAGACGGATGAAGTCAGGGTTCAGACAGCGGACGTCACTACAAAT
CTTCCGGACGTCAGGGACGCTGAAGATAAGAGCATCAGGGACACCGCAGT
GGCATATCCTGACGCCCAAAGTTTCCACCGCGCCATCTCCGCTATAGTCG
TGCTGGGGCAGTGCTTCGGTCTCCTGCCTGTGCATGGCGTAACGGCGTCA
AGCGCGCAAGGTCTCAGCACTGCAGCCATGGAGGTTTATTGTACTCTAAC
C

**Amino Acid Sequence**

MAKQTHVEVRRVLRPRDAVHQTPGRWGHKDAPWNTETDEVRVQTADVTTN
LPDVRDAEDKSIRDTAVAYPDAQSFHRAISAIVVLGQCFGLLPVHGVTAS
SAQGLSTAAMEVYCTLT

***Odontotermes formosanus* Gustatory receptor 9 (*OforGR9*) (**OR651384)

**Nucleotide Sequence**

ATGTCCATGAATCGGCCAGTGATATCCGTCCTTGGTTTCGTGACCATCAA
TAAAGGAACGGTAAAATCTTATGCGTCCACACTGGTGACCTACATGATAG
TCCTGTTGCAGTTCAGCATGGGACAGCATAAGCCTGACAATAACTGCAAT
GTGCTGCAGAACACAACAGAGACAAACTGA

**Amino Acid Sequence**

MSMNRPVISVLGFVTINKGTVKSYASTLVTYMIVLLQFSMGQHKPDNNCN
VLQNTTETN*

***OforSNMPs***

***Odontotermes formosanus* Sensory neuron membrane protein 1 (*OforSNMP1*) (**OR651358**)**

**Nucleotide Sequence**

ATGTTCGCCTTGGGTTCAGGAGTTGGATTCTACGGTTTCCCGTCTCTAAT
CCGCAGCCAGATAGCATCAAATTTAGCTCTGAAGAAGGGATCTGATTTAA
GGAAACTGTGGTCCAAGATTCCTGATGGGATTGACTTCAAAATATATATG
TTCAATATAACAAATCCAATGGACGTACAAGCAGGAAAGAAACCAATAGT
GACCGAAATTGGACCATATTTCTATGAAGAATACAAAGAGAAGCTAGACC
TCAAAGACCACAATGAAGACGATACAGTTTCATTCAATCCACGAGATTAT
TTTATATTCAAGAGAGAGAAATCTGGAGGTTTAACTGGAGATGAAATCAT
CACCATACCACATATGCCTATACTGGCAATGGCACTGGCAGTGGAGCGCG
AAAAGCCAGCGGCCCTGAAGCTAATCAACAAGGCAATACCTCACATCTTC
GGGCATCCGACCTCGGTCTTCTTAACGGCGCCTGTGAAGAACATTCTGTT
CGATGGGATTCCCCTGTACTGCAACGTCACAGATTTTTCCGCCAAAGCCA
TTTGCTCAGAAATAAGGAAAAACGACAAAAACTTTCTGAAATTAGGTGAA
GATATCTTCGGGTTTTCATTCTTCGGAACGAAAAACAACAGCGCTGGAGG
AAGGTTCCGCGTGAAACGTGGGATTCAGGATATTAAGGAGGTGGGGCGAG
TTGTGGAGTACGAAGGGCATAAACAACTGTCAGTCTACGATGGAGAAGAG
TGTAACAAGTTCAGAGGAACGGACTCCACCATCTTCGCGCCCTTCCTTAC
TCCAAGCGACAAGATCGAAGCTTTTGCTCCCGACCTCTGCAGGTCTATAG
GGGCTGTGTACAAGGAGTCGATCGTGTACAAAGGTATACACAGCTATAGT
TACGGCGCCGATTTCGGCGACATGTCTACCGACCCGGAACTCAAGTGTTT
CTGTACGACTCCAACCACTTGCATGAAGAAAGGCATTCACGACCTGACGA
GATGTACAGGGGCACCCCTCATGGCGTCACTTCCTCACTTCTACGACGCC
GCAATGGAGTATCAGACGGGAGTGATCGGCCTCAACCCTTCGAAAGAGAA
ACATGAGATACTGATGGTATTCGAACCGTTGACGTCCACACCTCTGGTTG
GATACAAGAGACTGCAGTTCAACATAGATGTTCATGCTATTGACAAGATT

GACCTCATGAAGGATATTCCCACAGTACTTCTCCCCGTATTGTGGGTGCA
AGAGGGAATGGAACTGAAACAAGAGTACCTGGACAAGGTAGCCAGCATCT
TCAAGATCATAGGCGCAGTGGACGTGGTCAAGTGGATCCTGATGGTGTTA
GGAGGAGGATGTGGTGCAGCAGGCGCAATCTTAGGGTACCGCAAGAAGTC
AAACGAAATGGACGTCGATGTGTCACCAAGTGTGCCGAAGAAACCAGCAG
GAATCACACCTCTGGAGGTGCAAACGCTACCCAGATACTGA

**Amino Acid Sequence**

MFALGSGVGFYGFPSLIRSQIASNLALKKGSDLRKLWSKIPDGIDFKIYM
FNITNPMDVQAGKKPIVTEIGPYFYEEYKEKLDLKDHNEDDTVSFNPRDY
FIFKREKSGGLTGDEIITIPHMPILAMALAVEREKPAALKLINKAIPHIF
GHPTSVFLTAPVKNILFDGIPLYCNVTDFSAKAICSEIRKNDKNFLKLGE
DIFGFSFFGTKNNSAGGRFRVKRGIQDIKEVGRVVEYEGHKQLSVYDGEE
CNKFRGTDSTIFAPFLTPSDKIEAFAPDLCRSIGAVYKESIVYKGIHSYS
YGADFGDMSTDPELKCFCTTPTTCMKKGIHDLTRCTGAPLMASLPHFYDA
AMEYQTGVIGLNPSKEKHEILMVFEPLTSTPLVGYKRLQFNIDVHAIDKI
DLMKDIPTVLLPVLWVQEGMELKQEYLDKVASIFKIIGAVDVVKWILMVL
GGGCGAAGAILGYRKKSNEMDVDVSPSVPKKPAGITPLEVQTLPRY*

***Odontotermes formosanus* Sensory neuron membrane protein 2 (*OforSNMP2*)** (OR651359)

**Nucleotide Sequence**

ATGAAGAAAGTGTCACTGAGGAATCAGTCCTCCGTATGGCTGGTCCTGTC
TGGGATGACCATCTTCGTGCTAGGGGCCGTGCTGGGATGGTATGGGTTCC
CGGCGTTAATAAGAAGCCAAATTACTGCTAACCTTGAGTTGAAGGAAGGA
GCTGAGAGAAGGGAAATTTGGGAAAGGGTTCCTTATCCGATGGATTTCAA
GATATACTTATTCAACGTAACAAACCCGATGGATGTTCAGAAGGGAGCAA
CGCCTGTCGTTCAGGAAGTGGGGCCGTACTGTTACAAGGAAGACAAAGAG
AAGGTCAATATTGTGGATCATGAAGACGATGATACAGTGTCCTTCAACTT
GAAAGACACGTGGTATTTCAACAAGGATGAGTCGGGCACCCTTACTGGCG
AAGAGAACATCACCATTCCGAATGTACTACTTCTGGGTATGGTTTTGACG

GCACAGCGCGAGCAGCCCATAGCCCTGAAGCTGATCAACACAGCCATCCC
ACACATCTTCGACAACCCGAACTCTGTATTCGTCACAGCCCCAGCAAAAA
ATTTGCTGTTCGAAGGCGTCGCGTTCAATTGCACATCCAGTGACTTCTCT
ACGAAGGCGGTCTGCTCAGAATTGAAAAAGAGAGCCCATAATTTCCACAG
AATCAGCGAAGACATCTACACGTTTTCTATTTTCGGATTCAAAAATGGCA
CTGTGCGGGAGAGGTTTGAGGTAAAGAGAGGAATGGAAGACATAAAGGAT
TTAGGCAAAATGGTGGAATTCAAGGATCAGAAAGTACTGACTGTCTGGGA
CGGAGAAGAGTGCAATGCGCTCAGAGGAACAGATTCCACCATCTTTCCTC
CCTTCCTCACAAAGAAAGACAAGATTGAAGGTTTCATTCCAGACATGTGC
AGGGCATTAGTGGCGGAGTACCAGTACGCCACAACGTACAGAGGAATCCG
GTCTTACAAATACAGCGCAGACTTGGGGGACACATCGACTGACCCGGAAC
TCGGGTGTTACTGCAGGACGCCAACTACGTGTCTCAAGAAAGGGGTTCAC
GATGTCTCGCGGTGTGCAGGGTACCCCGTCGTGATGTCACTGCCACACTT
CTACCTGGCTGACGACGAGTACCTGGATGGCGTAGTTGGCCTGAACCCTA
CACAGGAGAAGCACGAGGTCACTTTGCTCTTCGAACCGCTGACAGCTACA
CCACTTGAAGCTTACAACAGACTGCAGCTGAACATCCCCCTGCACCGCAC
CGACAGCATCGACCTACTGAAGAACATCAAATCAACGCTTCTTCCTATCT
TGTGGATGCAGGAGAACATGGAGCTCCATCAAGAGTACGTTGACAAGATT
TTGGACCTCTTCCTTATAATCAGCATAATGGGCGCCATGAAATGGATAAT
GGTAGCTGTTGGAGGAGTTCTCACTGCCACTGGATTCTTCTTCATTATGC
GGAGAAAGAACGATCATACTGTCATCACAGAATTACCACCCATCGCCTTA
AAACCCACAGGGAAATCGGCCAAGACGGAGCATGTTATCGAAAAATAG

**Amino Acid Sequence**

MKKVSLRNQSSVWLVLSGMTIFVLGAVLGWYGFPALIRSQITANLELKEG
AERREIWERVPYPMDFKIYLFNVTNPMDVQKGATPVVQEVGPYCYKEDKE
KVNIVDHEDDDTVSFNLKDTWYFNKDESGTLTGEENITIPNVLLLGMVLT
AQREQPIALKLINTAIPHIFDNPNSVFVTAPAKNLLFEGVAFNCTSSDFS
TKAVCSELKKRAHNFHRISEDIYTFSIFGFKNGTVRERFEVKRGMEDIKD

LGKMVEFKDQKVLTVWDGEECNALRGTDSTIFPPFLTKKDKIEGFIPDMC

RALVAEYQYATTYRGIRSYKYSADLGDTSTDPELGCYCRTPTTCLKKGVH

DVSRCAGYPVVMSLPHFYLADDEYLDGVVGLNPTQEKHEVTLLFEPLTAT

PLEAYNRLQLNIPLHRTDSIDLLKNIKSTLLPILWMQENMELHQEYVDKI

LDLFLIISIMGAMKWIMVAVGGVLTATGFFFIMRRKNDHTVITELPPIAL

KPTGKSAKTEHVIEK*

***Odontotermes formosanus* Sensory neuron membrane protein 3 (*OforSNMP3*) (partial) (**OR651360)

**Nucleotide Sequence**

ATGCCTGTGGCGGTGTTCTTCAATGTCTACCTATTCAACGTAACCAATCC

TGATGAAGTGCAGAAAGGAGCCAATCCAGTCGTGAAGGAAATCGGACCTT

ATGTATATGATGAGTACCGAGAGAAATTCGACATCGAAGACAAAGGTGAT

GGCACTCTTTCCTACTTACAAAACTCTACATTTTATTTCAACAAGGAAAA

GTCGGGGAACCTCTCAGAAGATGACGTAGTTACTGTACTGAACTTTGCGC

TCGTGGGTACGGTCTTAAAGGCACAGAGATACCTTCCTTCCTCCATGACA

CTAGATGGTGTTTTTAAGGAGATATTCCACGGGTCAGAGAACGTATTTCA

CACTGCCACAGTGGGGGATCTGGTTTGGCGTGGGGTAAAAGTCGTGACTT

GCACGGAGACAAATAAGACGTCGCCTGAAGCTTCGATGATCTGCAGTGTA

CTGAAAAGCCTACTGCCGGCTATGATGACTGAACATGAACCAGGGATCTT

CAAGATGGCGTATTTTCGTTACAAGAACAACACAAATGATGGTCGATACC

GCATTAACAGCGGAATTTCG

**Amino Acid Sequence**

MPVAVFFNVYLFNVTNPDEVQKGANPVVKEIGPYVYDEYREKFDIEDKGD

GTLSYLQNSTFYFNKEKSGNLSEDDVVTVLNFALVGTVLKAQRYLPSSMT

LDGVFKEIFHGSENVFHTATVGDLVWRGVKVVTCTETNKTSPEASMICSV

LKSLLPAMMTEHEPGIFKMAYFRYKNNTNDGRYRINSGIS

***Odontotermes formosanus* Sensory neuron membrane protein 4 (*OforSNMP4*) (partial) (**OR651361)

**Nucleotide Sequence**

ATGTGTTTAGGTGTGTCTGCAATCTATTCCTTGCCACATTTCTACAACGC

TTCTCCTGACTACCAACAGTACATCCAGGGTTTGAATCCAAACAGGAAGG

AACATGAGACATTCCTGTATATTGAACCGGAAACCGGTACCGTTCTCCGG

GGGTTCAAGCGGATGCAGATGAATATATTCCTTTCTAAAACGGATGTACT

AAGCTCCCTAAATAATGTGTCCGAAGGTCTCTTTCCTGTCTTCTGGGTTG

AAGAGGGCATTGAACTGGATGACAAGGATCTCACTCCAATTCACCGTCTG

TATACACTCATGTATGCCTTCGCCATTATCAGGTGGTTACTTGTAGCAGC

TGGC

**Amino Acid Sequence**

MCLGVSAIYSLPHFYNASPDYQQYIQGLNPNRKEHETFLYIEPETGTVLR

GFKRMQMNIFLSKTDVLSSLNNVSEGLFPVFWVEEGIELDDKDLTPIHRL

YTLMYAFAIIRWLLVAAG
